# Proton pump inhibitors are not associated with an increased risk of *Clostridioides difficile* infection: a systematic review and meta-analysis of randomized controlled trials

**DOI:** 10.1080/19490976.2025.2562341

**Published:** 2025-10-05

**Authors:** Diana-Elena Floria, Mahmoud Obeidat, Szilárd Váncsa, Sarolta Beáta Kávási, László Földvári-Nagy, Péter Hegyi, Dániel Sándor Veres, Vasile-Liviu Drug, Bálint Erőss

**Affiliations:** a Centre for Translational Medicine, Semmelweis University, Budapest, Hungary; b Grigore T. Popa University of Medicine and Pharmacy, Iași, Romania; c Institute of Pancreatic Diseases, Semmelweis University, Budapest, Hungary; d Department of Surgery, Toldy Ferenc Hospital, Cegléd, Hungary; e Department of Morphology and Physiology, Faculty of Health Sciences, Semmelweis University, Budapest, Hungary; f Institute for Translational Medicine, Medical School, University of Pécs, Pécs, Hungary; g Department of Biophysics and Radiation Biology, Semmelweis University, Budapest, Hungary

**Keywords:** Infectious colitis, enterocolitis, acid-suppression, pantoprazole, omeprazole, esomeprazole, lansoprazole

## Abstract

Observational studies reported conflicting results regarding the association between proton pump inhibitors (PPIs) and intestinal dysbiosis. We assessed the risk of enteric infections, including *Clostridioides difficile* infection (CDI), and small intestinal bacterial overgrowth (SIBO) among PPI users compared to non-users in randomized controlled trials (RCTs). A systematic search was conducted on April 15th, 2025 (CRD42023403322). Eligible RCTs compared adults treated with PPIs versusplacebo or alternative therapies. Risk ratios (RR) with corresponding 95% confidence interval (CI) were calculated using random effects models. Eight RCTs with 29,880 participants reported CDI rates. No significant difference was observed between PPI users and non-users (RR = 1.19, 95% CI: 0.75; 1.89). Four RCTs totaling 27,254 participants compared PPIs to H2-receptor blockers, showing no significant difference in CDI risk (RR = 0.72, 95% CI: 0.49; 1.07). Similarly, three RCTs (1,645 participants) comparing PPIs with potassium-competitive acid blockers found no significant difference (RR = 1.23, 95% CI: 0.43; 3.55). The qualitative synthesis found that other enteric infections and SIBO may be more common in PPI users. Data from RCTs suggest that PPIs do not seem to increase the risk of CDI compared to placebo and other acid-suppressive medications.

## Introduction

Proton pump inhibitors (PPIs) rank among the most widely prescribed medications globally, and their usage is consistently on the rise,^1,^^2^ Data from a systematic literature review found that almost one in four adults were prescribed a PPI and 25% remained on acid-suppressive therapy for more than a year, with nearly one-third continuing treatment beyond 3 y.^3^ However, recent findings indicate that in over two-thirds of ambulatory patients, PPI use may be inappropriate, with no clearly documented indication for ongoing therapy.^1,^^2^^,^^4^

Although generally considered a safe medication, PPIs have been reported to be associated with certain serious side effects, including an increased risk of intestinal dysbiosis.^5^ Previous meta-analyses have investigated the link between PPI treatment and *Clostridioides difficile* infection (CDI), estimating up to twice as high odds in PPI users than in non-users.^6-9^ Proposed mechanisms explaining this association state that by lowering gastric acidity, PPIs may allow ingested bacteria to survive passage through the stomach and inhibit the normal conversion of salivary nitrite into reactive oxygen species that help suppress *Clostridioides difficile* spores.^10^ Following spore acquisition, outcomes can range from transient asymptomatic colonization to severe, life-threatening disease, including diarrheal syndrome, ileus, toxic megacolon, and even colonic perforation.^11^ It represents a significant challenge in clinical practice, as it remains the leading cause of hospital-acquired infections, responsible for up to 30,000 deaths in the United States annually.^12,^^13^

Treatment with PPIs has also been hypothesized to increase the risk for developing small intestinal bacterial overgrowth (SIBO).^14^ This is characterized by excessive bacterial proliferation in the small intestine, leading to various digestive symptoms, including bloating, gas, distension, and diarrhea.^15^ It is estimated to be a prevalent, yet frequently overlooked condition, which can significantly impact the quality of life for those affected.^16^

Notably, these conclusions were based only on observational data, which showed significant unexplained heterogeneity. The latest guidelines for managing gastroesophageal reflux disease elaborated by the American College of Gastroenterology also advocate caution in interpreting these findings.^17^ Furthermore, results from recent large-scale trials do not seem to substantiate these associations,^18,^^19^ and none of the previously published systematic reviews addressed this association in the context of randomized controlled trials (RCTs).

Given the contradictory nature of the available data, we aimed to assess the risk of developing intestinal dysbiosis, more specifically CDI, other enteric infections, and SIBO, among adults receiving PPIs compared to non-users, based on data reported by RCTs.

## Methods

This systematic review and meta-analysis was performed in accordance with the Preferred Reporting Items for Systematic Reviews and Meta-Analyses (PRISMA) 2020 Guideline (Supplementary Table S1)^20^ and the recommendations of the Cochrane Handbook.^21^ We prospectively registered the study protocol on PROSPERO (CRD42023403322). This manuscript builds upon work previously shared as a preprint.^22^

### Eligibility criteria

Eligibility criteria were defined using the PICO framework (Population, Intervention, Comparator, Outcome). The population comprised adult patients (over 18 y old) without limitations based on gender or ethnicity. The intervention was treatment with PPIs (omeprazole, esomeprazole, pantoprazole, lansoprazole, dexlansoprazole, rabeprazole), regardless of dose, administration route, frequency, or treatment duration. The comparison group comprised non-users who received either a placebo or different pharmaceuticals (for comparative risk assessment, we included other acid-suppressive agents, such as histamine-2 receptor antagonists and potassium-competitive acid blockers; should any medication class demonstrate an elevated risk, specific recommendations favoring an alternative agent could be formulated). Rates of CDI, other enteric infections, and SIBO were the outcomes of interest. Only RCTs were considered eligible for inclusion.

We excluded studies involving pediatric populations and those lacking data on specified outcomes.

### Information sources

On April 15th, 2025, a systematic search was performed across three medical databases: MEDLINE (via PubMed), Embase, and the Cochrane Central Register of Controlled Trials (CENTRAL). No language or other restrictions were applied. No filters were used during the search.

### Search strategy

The search key consisted of two domains: the first pertaining to PPIs and the second to the concept of randomization. For the detailed search strategy, see Supplementary material Table S2.

### Selection process

Articles identified by the systematic search were imported into a reference management program (EndNote 20, Microsoft, USA). Duplicate items were removed manually and with automated methods by comparing authors, titles, and publication years. Two reviewers (D.E.F. and S.B.K.) independently screened and selected, initially assessing titles and abstracts, followed by a review of full texts. Cohen's kappa coefficient (*κ*) was calculated at both stages of selection to assess inter-reviewer agreement.^23^ In case of disagreement, consensus was reached through consultation with a third investigator (M.O.).

### Data collection process

Two authors (D.E.F. and S.B.K.) independently extracted relevant information from the studies included. A third reviewer (M.O.) resolved any disagreements. Data were manually compiled and entered into an Excel table (Office 365, Microsoft, USA) in preparation for statistical analysis.

### Data items

The following data were extracted from studies: name of first author, year of publication, digital object identifier (DOI), country, study design, number of involved centers, detailed description of the study population, basic demographics, details of intervention, and comparator (type of medication, dose, frequency of administration, and duration of treatment), follow-up time, data on rates of CDI, other enteric infections, and SIBO in the experimental and control groups, and details of definitions of outcomes of interest. Investigators attempted to contact authors to obtain missing data elements; no additional information was retrieved. Only information from the first study period was collected for cross-over trials, given the potential for a carry-over effect.

### Risk of bias and quality of evidence assessment

Two reviewers (D.E.F. and S.B.K.) independently evaluated the risk of bias using the Revised Cochrane Risk-of-bias Tool for Randomized Trials (RoB2).^24^ Any discrepancies were resolved by a third reviewer (M.O.).

The RoB2 tool consists of five main domains that assess potential sources of bias, including the randomization process, deviations from intended interventions, missing data, outcome measurement, and possible selection of reported results. On the basis of the answers to signaling questions, final assessments can be categorized as “Low risk of bias,” “Some concerns,” or “High risk of bias.”

We employed the Grading of Recommendations Assessment, Development and Evaluation (GRADE) approach, using the GRADEpro tool (software)^25^ to evaluate the quality of evidence. Primary determinants included study design, risk of bias, inconsistency, indirectness, and imprecision.

### Synthesis methods

The minimum number of studies needed to perform a meta-analysis was three. As we anticipated considerable between-study heterogeneity, a random-effects model was used to pool effect sizes.

The risk ratio (RR) with a 95% confidence interval (CI) was the main effect size measure. We extracted the total number of patients and events in each group from the publications to calculate the RRs, reporting the risk in the experimental group compared to the control group.

Statistical significance was attributed to results where the pooled CI did not encompass the null value. We summarized the findings of the meta-analysis in forest plots. Heterogeneity was described by the between-study variance (*τ*^2^) and Higgins and Thompson's *I*^*2*^ statistics.^26^ If feasible (given sufficient studies and manageable heterogeneity), prediction intervals were reported.

Potential publication bias was assessed by visual inspection of funnel plots and calculating the Harbord (modified Egger's) test *p*-value^27.^ We considered the possibility of small study bias if the *p*-value was below 10% while recognizing the limited utility of the test with fewer than ten studies.

Potential outlier publications were identified using various influence measures and plots following the recommendations of Harrer et al.^28^

All analyses were performed using R software,^29^ utilizing the *meta*^30^ package for basic calculations and plots and the *dmetar*^31^ package for additional influential analyses. For detailed information on calculations, data synthesis, publication bias assessment, and influential analyses, please refer to the detailed description of statistical methods in the Supplementary material – Statistical methods.

## Results

### Search and selection

**Figure 1.**
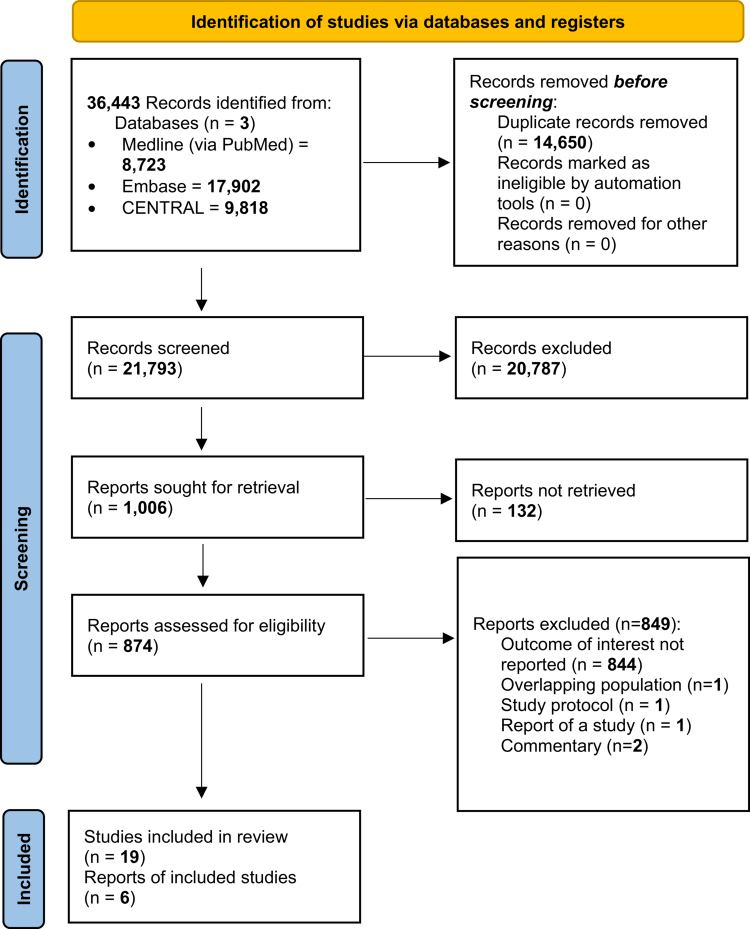
PRISMA flowchart detailing the search and selection process.

A systematic literature search resulted in 36,443 articles identified across three medical databases: 8723 entries in MEDLINE (via PubMed), 17,902 Embase, and 9818 CENTRAL. After duplicate removal, 21,793 articles remained for the title and abstract selection. After screening, we selected 1006 studies for full-text evaluation (*κ* = 0.94), with 132 unretrievable publications. Our broad search strategy was designed to maximize sensitivity and avoid missing potentially relevant trials. As a result, a large number of records (*n* = 20,787) were excluded during title and abstract screening because they were not RCTs or were unrelated to the elements of our PICO framework. Efforts were made to obtain the articles that could not be retrieved by reaching out to the respective journals and corresponding authors. Finally, 19 articles were considered eligible for inclusion (*κ* = 0.90) (Figure 1).

### Basic characteristics of the selected studies

The basic characteristics of the included studies are detailed in Table 1. All publications included were RCTs, of which 15 were multicenter studies. The authors compared the effect of PPIs to either a placebo, a histamine H2-receptor antagonist (H2RA) (cimetidine, famotidine), or a potassium-competitive acid blocker (vonoprazan). The articles eligible for inclusion used different dosages of PPIs and various treatment durations and administration frequencies. In terms of treatment indication, seven of the articles evaluated the effect of stress ulcer prophylaxis,^19,^^32-37^ four assessed patients on anticoagulants or antiplatelet medications for the prophylaxis of gastrointestinal bleeding,^18,^^38-40^ three investigated the healing of esophagitis,^41-43^ and the other two looked at the impact on peptic ulcer disease.^44,^^45^ The three remaining articles had various patient populations of interest, such as severe acute pancreatitis,^46^ individuals with hemochromatosis to assess the need for phlebotomy,^47^ and *Helicobacter pylori* eradication with or without acid suppression.^48^ In terms of outcomes, 15 of the studies reported data on the rates of CDI,^18,^^19,^^40,^^41,^^43,^^44,^^48,^^32-39^ four looked at other enteric infections,^18^^,^^39^^,^^42^^,^^47^ and two articles assessed the rates of SIBO.^45,^^46^

**Table 1.** Basic characteristics of included studies.

Author, year	Number of centers	Country	Age (years)		Number of female patients (%)		Intervention	Comparator	Indication	Outcome of interest	Follow-up time
			Intervention group	Comparator group	Intervention group	Comparator group					
Alhazzani et al., 2017^32^	10	Canada, Saudi Arabia, and Australia	Median 61.8 (IQR 48.4−73.5)	Median 55.3 (IQR 42.2−65.5)	44.9	40.5	Pantoprazole 40 mg iv once daily	Placebo	Stress ulcer prophylaxis	*Clostridioides difficile* infection	• PPI: median 12 d (IQR 8−23) • Placebo: median 8.5 d (IQR 6−18)
Bhatt et al., 2010^38^	393	Australia, Bulgaria, Canada, Chile, Czech Republic, France, Germany, Hungary, Italy, Mexico, Poland, Romania, Slovakia, Ukraine, USA	Median 68.5 (IQR 60.7−74.4)	Median 68.7 (IQR 60.6−74.7)	33.1	30.5	Omeprazole 20 mg po once daily	Placebo	Bleeding prophylaxis in patients with dual antiplatelet therapy	*Clostridioides difficile* infection	Median 106 d (IQR 55−166)
El-Kersh et al., 2018^33^	2	USA	Median 62 (IQR 49.5−68)	Median 58 (IQR 40.5−66.5)	45	40	Pantoprazole 40 mg iv once daily	Placebo	Stress ulcer prophylaxis	*Clostridioides difficile* infection	• PPI: median 6 d (IQR 4−9.5) • Placebo: median 7 d (IQR 3.5−11.5)
Krag et al., 2018^34^	33	Denmark, Finland, the Netherlands, Norway, Switzerland, and the United Kingdom	Median 67 (IQR 56−75)	Median 67 (IQR 55−75)	37	35	Pantoprazole 40 mg iv once daily	Placebo	Stress ulcer prophylaxis	*Clostridioides difficile* infection	90 d
Moayyedi et al., 2018^18^	580	International – 15 countries	Mean 67.6 (SD 8.1)	Mean 67.7 (SD 8.1)	22	21	Pantoprazole 40 mg po once daily	Placebo	Bleeding prophylaxis in patients with aspirin or anticoagulant treatment	*Clostridioides difficile* infection, Other enteric infections	Median 3.01 y (IQR 2.5−3.6)
Selvanderan et al., 2016^35^	1	Australia	Mean 52 (SD 18)	Mean 52 (SD 17)	36	33	Pantoprazole 40 mg iv once daily	Placebo	Stress ulcer prophylaxis	*Clostridioides difficile* infection	• PPI: median 16 d (IQR 8−31) • Placebo: median 18 d (IQR 9−25)
Cook et al., 2024^19^	68	Australia, Brazil, Canada, England, Kuwait, Pakistan, Saudi Arabia, USA	Mean 58.2 (SD 16.4)	Mean 58.3 (SD 16.4)	36.5	36.2	Pantoprazole 40 mg iv once daily	Placebo	Stress ulcer prophylaxis	*Clostridioides difficile* infection	90 d
Chen et al. (ABSTRACT), 2022^39^	Multicentric	NA	NA	NA	NA	NA	PPI	H2blocker/Control	Bleeding prophylaxis in patients with acute coronary syndrome	Enteric infections	NA
Wong et al., 2020^44^	1	China	Mean 67.6 (SD 16.3)	Mean 69.6 (SD 15.8)	31.6	44.7	Lansoprazole 30 mg po once daily	Famotidine 40 mg po once daily	Recurrent idiopathic ulcer bleeding prophylaxis	*Clostridioides difficile* infection	22 months
Young et al., 2020^36^	50	Australia, Canada, England, Ireland, New Zealand	Mean 58.6 (SD 17)	Mean 58.2 (SD 17.1)	36.2	36.1	PPI	H2blocker	Stress ulcer prophylaxis	*Clostridioides difficile* infection	90 d
Wee et al. (ABSTRACT), 2013^37^	1	USA	NA	NA	NA	NA	Pantoprazole 40 mg iv once daily	Famotidine 20 mg iv twice daily	Stress ulcer prophylaxis	*Clostridioides difficile* infection	• PPI: mean 6.7 d • H2Blocker: mean 6.5 d
Kawai et al., 2017^40^	104	Japan	Mean 68.3 (SD 9.06)	Provided for each Vonoprazan group	18	Provided for each Vonoprazan group	Lansoprazole 15 mg po once daily	Vonoprazan 10 mg po once daily/Vonoprazan 20 mg po once daily	Bleeding prophylaxis in patients with a personal history of peptic ulcer and low-dose aspirin treatment	*Clostridioides difficile* infection	2 y
Laine et al., 2023^41^	111	USA, Poland, Czech Republic, Hungary, Bulgaria, and United Kingdom	Mean 51.7 (SD 14.1)	Mean 51 (SD 13.4)	56.3	49.8	Lansoprazole 30 mg po once daily	Vonoprazan 20 mg po once daily	Healing of Erosive Esophagitis	*Clostridioides difficile* infection	8 weeks
Uemura et al., 2025^43^	36	Japan	Mean 61.5 (SD 12.2)	Mean 60.4 (SD 11.8)	38.8	28.1	Lansoprazole 30 mg po once daily	Vonoprazan 20 mg po once daily	Maintenance treatment for Erosive Esophagitis	*Clostridioides difficile* infection	5 y
De Boer et al., 1995^48^	1	Netherlands	Mean 51.7	Mean 50.9	37.04	31.48	Triple therapy (Bismuth, Tetracycline, Metronidazole) + Omeprazole 20 mg po twice daily	Triple therapy alone (no PPI)	*Helicobacter pylori* eradication with or without acid suppression	*Clostridioides difficile* infection	4−6 weeks
Ashida et al., 2015^42^	66	Japan	Mean 55.8 (SD 13.92)	Provided for each Vonoprazan group	29.3	Provided for each Vonoprazan group	Lansoprazole 30 mg po once daily	Vonoprazan 5 mg po once daily/Vonoprazan 10 mg po once daily/Vonoprazan 20 mg po once daily/Vonoprazan 40 mg po once daily	Healing of Erosive Esophagitis	Other enteric infections	8 weeks
Vanclooster et al., 2017^47^	2	Netherlands, Belgium	Mean 57.53 (SD 6.51)	Mean 53 (SD 10.5)	33	20	Pantoprazole 40 mg po once daily	Placebo	Patients with hemochromatosis, assessing the need for phlebotomy	Other enteric infections	12 months
Ma et al., 2020^46^	1	China	Mean 46.12 (SD 11.14)	Mean 44.55 (SD 9.29)	37	50	Esomeprazole 40 mg iv once daily	No PPI (conventional treatment)	Severe acute pancreatitis	SIBO	7 d
Thorens et al., 1997^45^	NA	Switzerland	NA	NA	NA	NA	Omeprazole 20 mg po once daily	Cimetidine 800 mg po once daily	Peptic disease	SIBO	28 d

Abbreviations: iv, intravenously; po, per orally; NA, not available; IQR, interquartile range; SD, standard deviation; PPI, proton pump inhibitor; USA, United States of America.

The daily dose equivalents of PPIs vary among different pharmaceutical agents, as their acid-suppression potency differs widely. When standardizing the relative acid-suppression potencies of individual PPIs based on their effects on mean 24-h intragastric pH, omeprazole serves as the reference with an “omeprazole equivalent” (OE) of 1.00. The estimated relative potencies for standard doses are as follows: pantoprazole at 0.23 OEs, lansoprazole at 0.90 OEs, omeprazole at 1.00 OE, esomeprazole at 1.60 OEs, and rabeprazole at 1.82 OEs.^49,^^50^

For the detailed definitions and criteria used for ascertaining CDI in each of the included studies, see Supplementary material, Table S3.

### Quantitative synthesis

#### Risk of developing CDI in PPIs versus placebo

Eight RCTs^18,19,32–35,38,39^ totaling 29,880 patients (14,946 in the PPI group, 14,934 in the placebo group) investigated the rates of CDI in individuals receiving treatment with PPIs compared to placebo. Six of the studies^19,32–35,39^ included hospitalized patients receiving short courses of PPIs as stress ulcer prophylaxis,^19,^^32-35^ and as bleeding prophylaxis in acute coronary syndrome.^39^ Two of them assessed outpatients undergoing long-term acid-suppressive treatment as bleeding prophylaxis in patients with antithrombotic therapy.^18,^^38^

**Figure 2.**
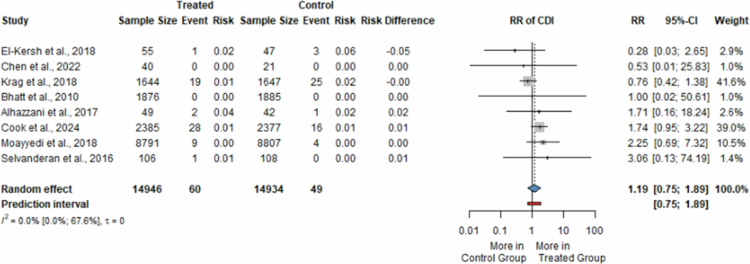
Forest plot showing the risk of developing *Clostridioides difficile* infection in patients receiving proton pump inhibitors compared to placebo (RR, risk ratio; CI, confidence interval; CDI, *Clostridioides difficile* infection).

Our analysis found no significant difference in the risk of developing CDI between the two study groups (RR = 1.19, 95% CI: 0.75; 1.89, *I*^*2*^ = 0%) (Figure 2). Low incidence rates of CDI were reported, with 60 cases in 14,946 participants undergoing acid-suppressive therapy with PPIs (0.40%). To a similar extent, 49 cases were diagnosed out of 14,934 subjects (0.33%) receiving placebo.

Additional subgroup analyses were intended based on patient population, treatment duration, and follow-up time; however, insufficient data were available for a meta-analysis (Supplementary material Figures S1–S3).

#### Risk of developing CDI in PPIs versus H2-receptor antagonists

Four studies^36^^,^^37^^,^^39^^,^^44^ compared 27,254 patients treated with PPIs to H2RA (13,658 in the PPI group, 13,596 in the H2RA). Three of the trials included hospitalized patients receiving short-term PPI treatment for stress ulcer prophylaxis,^36,^^37^ or bleeding prophylaxis in acute coronary syndrome.^39^ One study evaluated outpatients undergoing long-term therapy as bleeding prophylaxis for recurrent idiopathic ulcers. ^44^

**Figure 3.**
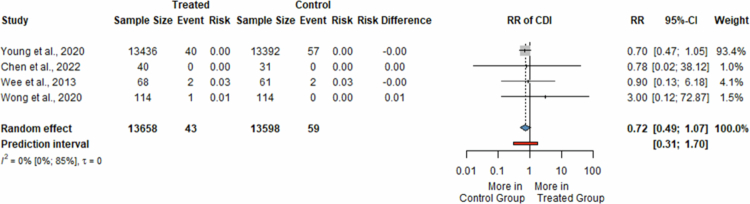
Forest plot showing the risk of developing *Clostridioides difficile* infection in patients receiving proton pump inhibitors compared to H2 receptor antagonists (RR, risk ratio; CI, confidence interval; CDI, *Clostridioides difficile* infection).

The meta-analysis did not find a significant difference in the risk of developing CDI between the two study groups (RR = 0.72, 95% CI: 0.49; 1.07, *I*^*2*^ = 0%) (Figure 3). The incidence rates of CDI were low, with 43 cases among 13,658 participants receiving PPIs (0.32%) and 59 cases out of 13,598 subjects (0.43%) receiving H2RA.

More detailed subgroup analyses were planned based on patient population, treatment duration, and follow-up time; however, there were insufficient data for further meta-analytical calculations (Supplementary material Figures S4–S6).

#### Risk of developing CDI in PPIs versus potassium-channel acid blockers

**Figure 4.**
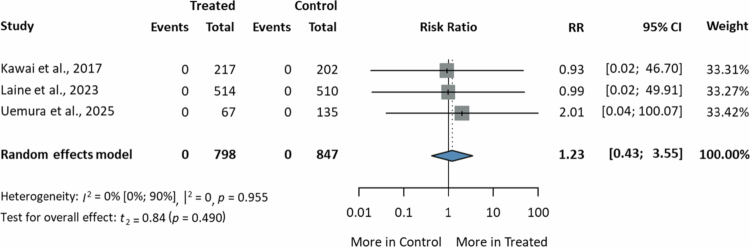
Forest plot showing the risk of developing *Clostridioides difficile* infection in patients receiving proton pump inhibitors compared to potassium-competitive acid blockers (RR, risk ratio; CI, confidence interval; CDI, *Clostridioides difficile* infection).

Three RCTs^40^^,^^41^^,^^43^ reported data on the incidence of CDI in individuals undergoing PPI therapy compared to vonoprazan. The study by Kawai et al.^40^ assessed 419 patients (217 in the PPI arm, 202 in the vonoprazan arm) with a personal history of peptic ulcer requiring chronic low-dose aspirin administration, with a follow-up time of 24 weeks. Laine et al.^41^ conducted a study on 1024 individuals (514 in the PPI group, 510 in the vonoprazan group) with erosive esophagitis, with an 8-week follow-up period. In the VISION trial conducted by Uemura et al.^43^ a total of 139 patients with erosive esophagitis who achieved mucosal healing following treatment with vonoprazan, along with 69 patients treated with lansoprazole, were maintained on long-term acid-suppressive therapy for a duration of 260 weeks. None of these trials reported any instances of CDI within their respective study populations. The meta-analytical calculations did not show a significant difference in the risk of developing CDI between the two study groups (RR = 1.23, 95% CI: 0.43; 3.55, *I*^2^ = 0%) (Figure 4).

### Qualitative synthesis

#### Risk of other enteric infections in PPIs versus placebo

Three RCTs^18^^,^^39^^,^^47^ reported data on the other enteric infections in patients treated with PPIs compared to placebo. A study by Moayyedi et al.^18^ had a median follow-up time of around 3 y and reported low incidence rates of enteric infections. There were 119 cases diagnosed in 8791 (1.4%) individuals undergoing PPI treatment and 90 cases in 8807 (1%) subjects receiving placebo (RR = 1.33, 95% CI: 1.01–1.75). Vanclooster et al.^47^ included 15 patients in each study arm and reported only one instance of enteric infection in the PPI-treated group. Chen et al.^39^ assessed 61 patients, 40 receiving PPI therapy and 20 in the control arm, and reported no cases of enteric infection in the study population.

#### Risk of other enteric infections in PPIs versus H2-receptor antagonists

Only one study provided information on this outcome. Chen et al.^39^ conducted a comparative analysis involving 40 individuals receiving PPI treatment and 31 undergoing therapy with H2RA. No occurrences of enteric infections were observed in either study arm.

#### Risk of other enteric infections in PPIs versus potassium-channel acid blockers

A study by Ashida et al.^42^ totaling 732 participants compared the effects of lansoprazole to various doses of vonoprazan (5, 10, 20, and 40 mg orally daily) for the treatment of erosive esophagitis, with an 8-week follow-up period. Only one case of enteric infection occurred in the study arm, with 40 mg of vonoprazan daily.

#### Risk of SIBO in PPIs versus non-PPIs

Two RCTs^45^^,^^46^ investigated the rates of SIBO in subjects undergoing treatment with PPIs. Ma et al.^46^ studied the effect of acid suppression on duodenal microbiota in 66 patients with severe acute pancreatitis, comparing added esomeprazole to conventional therapy alone (33 in the PPI group and 33 in the conventional treatment group). Duodenal bacterial overgrowth was defined as >10^3^ colony-forming units per milliliter (CFU/mL). In aerobic cultures, 29 out of 33 patients receiving PPIs met this criterion versus only 15 in the conventional group (RR = 1.93, 95% CI: 1.30; 2.86). Anaerobic cultures showed 28 cases in the PPI group compared to 14 in the conventional group (RR = 2.95% CI: 1.31; 3.05). Using a >10^5^ CFU/mL threshold, we identified 20 cases in subjects undergoing PPI therapy, compared to 8 in the conventional treatment group in aerobic cultures (RR = 2.5, 95% CI: 1.28; 4.85). Similar results were found in anaerobic cultures, with 20 cases in the acid-suppressive group versus 7 in the conventional treatment group (RR = 2.86, 95% CI: 1.4; 5.83). Thorens et al.^45^ assessed 37 patients with peptic disease, randomly assigned to either omeprazole or cimetidine. The prevalence of duodenal bacterial overgrowth, defined as surpassing 10^5^ CFU/ml in aerobic cultures, was higher in individuals receiving PPIs compared to H2RA (7 out of 19 patients in the PPI study arm versus 3 out of 18 participants receiving cimetidine; RR = 2.21, 95% CI: 0.67; 7.25, *p* = 0.19).

### Risk of bias assessment

Concerns about the risk of bias were raised in most of the studies included, whereas some publications exhibited a high risk of bias. Notably, several studies lacked a pre-published protocol or statistical analysis plan, prompting concerns about the selection of reported results. Another critical issue was insufficient details on the definition and diagnosis of outcomes of interest. For a detailed assessment, see Supplementary material Figures S7–S9.

### Certainty of evidence

The use of the GRADE approach deemed the quality of evidence very low. This was mainly due to the increased indirectness of the evidence. The patient categories in the studies included varied widely, ranging from hospitalized subjects to outpatients, with different indications for acid-suppressive treatment. The studies included also used varying doses, administration routes, and treatment durations. Additionally, the follow-up time ranged widely, from days of hospital stay to 3 y of follow-up. For a detailed assessment, see Supplementary material Tables S4–S5.

### Heterogeneity and publication bias

As the total number of studies was low and the individual study CIs were wide, the assessment of between-study heterogeneity is rather uncertain. We did not perform Egger's test for small study publication bias as fewer than 10 studies were included for the meta-analytical calculations per outcome. Although, the visual inspection of the generated funnel plots did not reveal any sign of potential publication bias (see Supplementary material Figure S10).

## Discussion

PPIs rank among the most frequently prescribed medications worldwide, as recent epidemiological findings suggest that approximately one-quarter of adults use these acid-suppressing drugs, especially over 65 y of age.^3^ Their safety profile has generally been regarded as excellent; however, concerns have been raised regarding specific side effects associated with prolonged use.^17^ While many of these primarily involve minor risks, such as hypomagnesemia^51^^,^^52^ or vitamin B12 deficiency,^53^ there are also hypotheses regarding more clinically significant and serious manifestations. Among these, the increased risk of *Clostridioides difficile* infection stands out as particularly critical, given its potential for significant morbidity and mortality due to life-threatening complications. The incriminated mechanistic pathway for this association is based on lower gastric acid production, which enables ingested enteric pathogens to survive gastric passage and favors bacterial overgrowth in the upper gastrointestinal tract.^54^ This work focused on comprehensively assessing these risks in patients on PPI treatment compared to non-users in the context of RCTs.

A number of previously reported associations between PPI use and adverse effects have been derived from observational data. These types of studies fail to establish causality and are prone to bias, such as confounding by indication and protopathic bias.^55,^^56^ In the context of PPI use, prevalent biases may include the misclassification of patients who require PPIs due to pre-existing gastrointestinal conditions, which could lead to an overestimation of the association between PPI use and enteric infections. Additionally, residual confounding may arise from unmeasured factors that contribute to both PPI use and the risk of developing infections. Several associations identified by observational research are false, whereas the minority of those that are true are often overstated.^57^ Specialists advise that findings from observational studies should not be deemed credible unless RRs in cohort studies surpass two or three or odds ratios (ORs) in case-control studies exceed three or four.^57^

One of the most important outcomes of this study was the risk of developing CDI, given its significant morbidity, mortality, and major financial impact on healthcare systems worldwide.^58^ This presents a major challenge in clinical practice, as it continues to be the primary cause of hospital-acquired infections, responsible for up to 30,000 deaths each year in the United States.^12^ Potential complications associated with CDI include colectomies, recurrent CDI, hospital readmissions, and transfers to long-term care facilities.^58^ On the basis of pooled data from eight placebo-controlled RCTs totaling almost 30,000 participants, treatment with PPIs was not associated with an increased risk of acquiring CDI. A similar conclusion was reached by assessing four other RCTs comparing subjects receiving PPIs to H2RA, involving more than 27,000 patients. Additionally, treatment with PPIs did not seem to increase the risk of acquiring CDI when compared to newer acid-suppressive medications, such as potassium-competitive acid blockers. These findings stand in contrast to the conclusions of previously published meta-analyses, which estimated that patients receiving PPIs were up to twice as likely to develop CDI as those not using them.^6-9,59-64^ These results from previous works were based exclusively on observational data, with significant unexplained heterogeneity. The discrepancy between our findings and those of prior observational studies may be explained by several methodological and mechanistic factors. Observational studies are prone to confounding by indication and protopathic bias, since patients prescribed PPIs are often older, hospitalized, and exposed to antibiotics, which could increase CDI risk. In contrast, RCTs mitigate these biases through randomization and thorough patient selection. Furthermore, most RCTs assessed short-term or prophylactic PPI use, whereas observational cohorts often included long-term users, in whom chronic hypochlorhydria may have a greater impact on intestinal microbiota. Taken together, these considerations provide a biological and methodological rationale for why our RCT-based meta-analysis did not confirm the stronger associations reported in observational studies.

For other enteric infections, our systematic search identified one particularly representative high-quality randomized trial conducted by Moayyedi et al.^18^ Some strong points of this work are the high number of patients included and the long follow-up time, with a median of 3 y. This study investigated subjects with stable atherosclerotic vascular disease on antithrombotic medication, randomly assigned to PPIs or placebo. A statistically significant, modestly increased risk of enteric infections was seen in those allocated to PPIs compared to non-users. Notably, this observed risk was lower than that estimated by systematic reviews of observational studies.^65^

In our work, we also evaluated the occurrence of SIBO, a condition characterized by an abundance of bacteria in the small intestine, leading to gastrointestinal symptoms.^15^ Patients with SIBO report a variety of symptoms, including nausea, bloating, abdominal pain, and altered bowel transit ranging from diarrhea to constipation, all of which can adversely affect their quality of life.^15^^,^^16^ This condition can also cause malabsorption, potentially resulting in unintended weight loss, anemia, and deficiencies in fat-soluble vitamins.^15^ In terms of its diagnosis, small bowel aspirates and culture are conventionally deemed the gold standard method, but with controversial threshold values for defining a positive culture. The 2017 North American Consensus suggests a cut-off of 10^3^ CFU/mL in duodenal or jejunal aspirates as a diagnostic of SIBO.^66^ PPI use has been described as an independent risk factor for the occurrence of SIBO,^67^ with a meta-analysis of 19 observational studies including over 7000 patients reporting a potential threefold increase in the risk of SIBO (OR = 1.71; 95% CI: 1.20; 2.43).^14^ Two RCTs found significantly higher rates of SIBO in participants receiving PPI compared to conventional treatment without additional acid-suppression^46^ and H2RA.^45^ These studies employed varying threshold values to determine the presence of SIBO while including small numbers of participants. On the other hand, an extensive deep-sequencing study explored the impact of PPI use on small bowel microbiome. The findings showed that SIBO was not detected through either culture or sequencing methods, and no significant changes in microbial diversity were noted.^68^

### Strengths and limitations

This systematic review and meta-analysis comprehensively examined the association between PPI use and the risk of developing CDI, other enteric infections and SIBO. Results were derived only from RCTs, contributing to the reliability and validity of our findings. The use of placebo, H2RA, and potassium channel acid blockers as comparators provided additional insight, enabling a more nuanced evaluation of the safety profile. The substantial number of patients included further increased the statistical power. We adopted a rigorous methodological approach in this systematic review.

Despite the comprehensive nature of this work, certain limitations should be mentioned. None of the included studies were designed with enteric infections or SIBO as their primary outcomes. There were notable variations in treatment durations, doses, and follow-up times across the articles included. However, while variability may pose challenges, it can also contribute to robust and applicable findings when accompanied by strong statistical support, such as the tight confidence intervals and low heterogeneity.

Across included RCTs, CDI diagnosis was established using a range of methods (including enzyme immunoassays for toxin A/B, PCR-based detection, culture, and in some cases, colonoscopy-based diagnosis supported by histopathological confirmation). Variation in diagnostic sensitivity and specificity could have contributed to heterogeneity in reported CDI rates. However, as our analysis did not identify significant heterogeneity, this variability likely exerted only a limited effect on the pooled estimates, though it remains an important methodological consideration.

### Implications for practice and research

Translating scientific knowledge into community benefits is essential.^69^^,^^70^ Insights from this work will improve our understanding of the safety profile of PPIs. The risk of acquiring CDI associated with PPI use may be lower than previously thought. Therefore, in the presence of a valid clinical indication for acid suppression, concerns about an increased risk of CDI should not deter physicians from prescribing PPIs.

Given the widespread use of PPIs, there are significant opportunities for targeted deprescribing efforts in order to improve patient care and reduce unnecessary treatment. As highlighted by the American Gastroenterological Association, many patients continue PPI therapy without a valid clinical indication, recommending that all patients on PPI therapy undergo regular review of their indications for use. Deprescribing strategies include dose reduction, transition to on-demand therapy, or complete discontinuation, particularly in patients without high-risk conditions such as complicated gastroesophageal reflux disease, Barrett's esophagus, Zollinger-Ellison syndrome or eosinophilic esophagitis.^2^

We recognize the need for further studies to refine the existing understanding of PPI safety. More high-quality RCTs with appropriate sample sizes would be useful to facilitate precise conclusions. However, we do acknowledge the practical limitations and costs associated with conducting RCTs, particularly in the context of generic drugs. Observational studies, when designed and conducted rigorously, can serve as valuable alternatives to RCTs. By utilizing advanced statistical techniques such as propensity score matching, instrumental variable analysis, and adjustment for confounding variables, observational studies can approximate the robustness of RCT findings while reflecting real-world scenarios. Furthermore, leveraging large databases and real-world evidence allows for a more comprehensive understanding of the long-term effects and potential harms of these medications.

One factor that may contribute to discrepancies across studies is geographical variation in circulating *C. difficile* strains. Our review included patients from diverse international settings, with a relatively low overall incidence of CDI. Future investigations should incorporate geographic and strain-specific analyses to clarify these associations better.

Additional secondary outcomes associated with the use of these medications should be more thoroughly investigated, such as other various enteric infections and rates of SIBO.

## Conclusion

Based on data from RCTs, PPI treatment does not appear to significantly increase the already low incidence of CDI, whether compared to placebo or H2-receptor antagonists. However, given the limitations of available studies, including relatively short follow-up durations, heterogeneous populations, and variable diagnostic methods, a modest increase in CDI risk cannot be entirely excluded, and prompt caution interpretation is advised in interpreting the results. Other enteric infections and SIBO may be more common in patients on PPI treatment than non-users, but published data are limited. Clinicians should prescribe PPIs in the presence of a valid clinical indication, balancing benefits against potential harms.

## Supplementary Material

SUPPLEMENTARY MATERIAL

## Data Availability

Data sharing not applicable – Data sharing is not applicable to this article as no new data were analyzed in this study. All information used in this study can be collected from the full text and supplementary materials of the cited articles, which are publicly available.

## References

[1] Torres-Bondia F, de Batlle J, Galván L, Buti M, Barbé F, Piñol-Ripoll G. Evolution of the consumption trend of proton pump inhibitors in the Lleida health region between 2002 and 2015. BMC Public Health [Internet]. 2022;22(1):1–8. doi: 10.1186/s12889-022-13217-6. 35461252 PMC9035259

[2] Targownik LE, Fisher DA, Saini SD. AGA clinical practice update on de-prescribing of proton pump inhibitors: expert review. Gastroenterology [Internet]. 2022;162(4):1334–1342. doi: 10.1053/j.gastro.2021.12.247. 35183361

[3] Shanika LGT, Reynolds A, Pattison S, Braund R. Proton pump inhibitor use: systematic review of global trends and practices. Eur J Clin Pharmacol. 2023 Sep;79(9):1159–1172. doi: 10.1007/s00228-023-03534-z. 37420019 PMC10427555

[4] Rotman SR, Bishop TF. Proton pump inhibitor use in the U.S. ambulatory setting, 2002-2009. PLoS One. 2013;8(2):2002–2009. 10.1371/journal.pone.0056060 PMC3572154 23418510

[5] Kiecka A, Szczepanik M. Proton pump inhibitor-induced gut dysbiosis and immunomodulation: current knowledge and potential restoration by probiotics. Pharmacol Rep [Internet]. 2023;75(4):791–804. doi: 10.1007/s43440-023-00489-x. 37142877 PMC10159235

[6] Trifan A, Stanciu C, Girleanu I, Stoica OC, Singeap AM, Maxim R, Chiriac SA, Ciobica A, Boiculese L. Proton pump inhibitors therapy and risk of *Clostridium difficile* infection: systematic review and meta-analysis. World J Gastroenterol. 2017;23(35):6500–6515. doi: 10.3748/wjg.v23.i35.6500. 29085200 PMC5643276

[7] Cao F, Chen CX, Wang M, Liao HR, Wang MX, Hua SZ, Huang B, Xiong Y, Zhang J, Xu Y. Updated meta-analysis of controlled observational studies: proton-pump inhibitors and risk of *Clostridium difficile* infection. J Hosp Infect. 2018;98(1):4–13. doi: 10.1016/j.jhin.2017.08.017. 28842261

[8] D'Silva KM, Mehta R, Mitchell M, Lee TC, Singhal V, Wilson MG, D'Silva KM, McDonald EG. Proton pump inhibitor use and risk for recurrent *Clostridioides difficile* infection: a systematic review and meta-analysis. Clin Microbiol Infect [Internet]. 2021;27(5):697–703. doi: 10.1016/j.cmi.2021.01.008. 33465501

[9] Mehta P, Nahass RG, Brunetti L. Acid suppression medications during hospitalization as a risk factor for recurrence of *Clostridioides difficile* infection: systematic review and meta-analysis. Clin Infect Dis. 2021;73(1):E62–E68. doi: 10.1093/cid/ciaa545. 32386313 PMC8246810

[10] Cunningham R, Mustoe E, Spiller L, Lewis S, Benjamin N. Acidified nitrite: a host defence against colonization with *C. difficile* spores? J Hosp Infect. 2014;86(2):155–157. doi: 10.1016/j.jhin.2013.12.003. 24440371

[11] Feuerstadt P, Theriault N, Tillotson G. The burden of CDI in the United States: a multifactorial challenge. BMC Infect Dis [Internet]. 2023;23(1):1–8. doi: 10.1186/s12879-023-08096-0. 36882700 PMC9990004

[12] McDonald LC, Gerding DN, Johnson S, Bakken JS, Carroll KC, Coffin SE, Dubberke ER, Garey KW, Gould CV, Kelly C, et al. Clinical practice guidelines for *Clostridium difficile* infection in adults and children: 2017 update by the infectious diseases society of America (IDSA) and society for healthcare epidemiology of America (SHEA). Clin Infect Dis an Off Publ Infect Dis Soc Am. 2018;66(7):e1–e48. doi: 10.1093/cid/cix1085. PMC6018983 29462280

[13] Fu Y, Luo Y, Grinspan AM. Epidemiology of community-acquired and recurrent *Clostridioides difficile* infection. Ther Adv Gastroenterol. 2021;14:17562848211016248. 10.1177/17562848211016248. PMC8141977 34093740

[14] Su T, Lai S, Lee A, He X, Chen S. Meta-analysis: proton pump inhibitors moderately increase the risk of small intestinal bacterial overgrowth. J Gastroenterol. 2018;53(1):27–36. doi: 10.1007/s00535-017-1371-9. 28770351

[15] Pimentel M, Saad RJ, Long MD, Rao SSC. ACG clinical guideline: small intestinal bacterial overgrowth. Am J Gastroenterol. 2020;115(2):165–178. doi: 10.14309/ajg.0000000000000501. 32023228

[16] Liébana-Castillo AR, Redondo-Cuevas L, Nicolás Á, Martín-Carbonell V, Sanchis L, Olivares A, Grau F, Ynfante M, Colmenares M, Molina ML, et al. Should we treat sibo patients? Impact on quality of life and response to comprehensive treatment: a real-world clinical practice study. Nutrients. 2025;17:1251. doi: 10.3390/nu17071251. 40219008 PMC11990593

[17] Katz PO, Dunbar KB, Schnoll-Sussman FH, Greer KB, Yadlapati R, Spechler SJ. ACG clinical guideline for the diagnosis and management of gastroesophageal reflux disease. Am J Gastroenterol. 2022;117(1):27–56. doi: 10.14309/ajg.0000000000001538. 34807007 PMC8754510

[18] Moayyedi P, Eikelboom JW, Bosch J, Connolly SJ, Dyal L, Shestakovska O, et al. Safety of proton pump inhibitors based on a large, multi-year, randomized trial of patients receiving rivaroxaban or aspirin. Gastroenterology. 2019;157(3):682–691.e2. 31152740 10.1053/j.gastro.2019.05.056

[19] Cook D, Deane A, Lauzier F, Zytaruk N, Guyatt G, Saunders L, Hardie M, Heels-Ansdell D, Alhazzani W, Marshall J, et al. Stress ulcer prophylaxis during invasive mechanical ventilation. N Engl J Med. 2024;391(1):9–20. doi: 10.1056/NEJMoa2404245. 38875111

[20] Page MJ, McKenzie JE, Bossuyt PM, Boutron I, Hoffmann TC, Mulrow CD, Shamseer L, Tetzlaff JM, Akl EA, Brennan SE, et al. The PRISMA 2020 statement: an updated guideline for reporting systematic reviews. BMJ. 2021;372:n71. doi: 10.1136/bmj.n71. 33782057 PMC8005924

[21] Higgins J, Thomas J, Chandler J, Cumpston M, Li T, Page M, et al. Cochrane handbook for systematic reviews of interventions version 6.3. 2022. (updated February 2022). London, UK: Cochrane.

[22] Floria D-E, Obeidat M, Váncsa S, Kávási SB, Földvári-Nagy L, Hegyi P, Veres DS, Drug VL, Eross B. Proton pump inhibitors are not associated with an increased risk of Clostridioides difficile infection: a systematic review and meta-analysis of randomized controlled trials [Internet]. New York, USA: SSRN. 10.1080/19490976.2025.2562341 PMC12502825 41047657

[23] Cohen J. A coefficient of agreement for nominal scales. Educ Psychol Meas [Internet]. 1960;20(1):37–46. doi: 10.1177/001316446002000104.

[24] Sterne JAC, Savović J, Page MJ, Elbers RG, Blencowe NS, Boutron I, Cates CJ, Cheng H, Corbett MS, Eldridge SM, et al. RoB 2: a revised tool for assessing risk of bias in randomised trials. BMJ. 2019;366:l4898. doi: 10.1136/bmj.l4898. 31462531

[25] Guyatt G, Oxman AD, Akl EA, Kunz R, Vist G, Brozek J, Norris S, Falck-Ytter Y, Glasziou P, deBeer H. GRADE guidelines: 1. Introduction-GRADE evidence profiles and summary of findings tables. J Clin Epidemiol. 2011;64(4):383–394. doi: 10.1016/j.jclinepi.2010.04.026. 21195583

[26] Higgins JPT, Thompson SG. Quantifying heterogeneity in a meta-analysis. Stat Med. 2002;21(11):1539–1558. doi: 10.1002/sim.1186. 12111919

[27] Harbord RM, Harris RJ, Sterne JAC. Updated tests for small-study effects in meta-analyses. Stata J [Internet]. 2009;9(2):197–210. doi: 10.1177/1536867X0900900202.

[28] Harrer M, Cuijpers P, Toshi F, Ebert DD. Doing meta-analysis with R: a hands-on guide, 1st ed. Boca Raton, FL: Chapman and Hall/CRC; 2021.

[29] Team RC. R: a language and environment for statistical computing. Vienna, Austria: R Foundation for Statistical Computing; 2023.

[30] Schwarzer G. Meta: general package for meta-analysis. 2022. Vienna, Austria: The R Foundation for Statistical Computing.

[31] Pim C, Furukawa T, Ebert DD. Dmetar: companion R package for the guide doing meta-analysis in R. 2022. Vienna, Austria: The R Foundation for Statistical Computing.

[32] Alhazzani W, Guyatt G, Alshahrani M, Deane AM, Marshall JC, Hall R, Muscedere J, English SW, Lauzier F, Thabane L, et al. Withholding pantoprazole for stress ulcer prophylaxis in critically ill patients: a pilot randomized clinical trial and meta-analysis. Crit Care Med. 2017;45(7):1121–1129. doi: 10.1097/CCM.0000000000002461. 28459708

[33] El-Kersh K, Jalil B, McClave SA, Cavallazzi R, Guardiola J, Guilkey K, Persaud AK, Furmanek SP, Guinn BE, Wiemken TL, et al. Enteral nutrition as stress ulcer prophylaxis in critically ill patients: a randomized controlled exploratory study. J Crit Care [Internet]. 2018;43:108–113. doi: 10.1016/j.jcrc.2017.08.036. 28865339

[34] Krag M, Marker S, Perner A, Wetterslev J, Wise MP, Schefold JC, Keus F, Guttormsen AB, Bendel S, Borthwick M, et al. Pantoprazole in patients at risk for gastrointestinal bleeding in the ICU. N Engl J Med. 2018;379(23):2199–2208. doi: 10.1056/NEJMoa1714919. 30354950

[35] Selvanderan SP, Summers MJ, Finnis ME, Plummer MP, Ali Abdelhamid Y, Anderson MB, Chapman MJ, Rayner CK, Deane AM. Pantoprazole or placebo for stress ulcer prophylaxis (pop-up): randomized double-blind exploratory study. Crit Care Med. 2016;44(10):1842–1850. doi: 10.1097/CCM.0000000000001819. 27635481

[36] Young PJ, Bagshaw SM, Forbes AB, Nichol AD, Wright SE, Bailey M, Bellomo R, Beasley R, Brickell K, Eastwood GM, et al. Effect of stress ulcer prophylaxis with proton pump inhibitors vs histamine-2 receptor blockers on in-hospital mortality among icu patients receiving invasive mechanical ventilation: the peptic randomized clinical trial. JAMA. 2020;323(7):616–626. doi: 10.1001/jama.2019.22190. 31950977 PMC7029750

[37] Wee B, Liu C, Cohen H, Kravchuk S, Reddy K. IV famotidine vs. IV pantoprazole for stress ulcer prevention in the ICU: a prospective study. Crit Care Med [Internet]. 2013;41(12 Suppl 1):A181. Available from: http://ovidsp.ovid.com/ovidweb.cgi?T=JS&PAGE=reference&D=emed11&NEWS=N&AN=71533909.

[38] Bhatt DL, Cryer BL, Contant CF, Cohen M, Lanas A, Schnitzer TJ, Shook TL, Lapuerta P, Goldsmith MA, Laine L, et al. Clopidogrel with or without omeprazole in coronary artery disease. N Engl J Med. 2010;363(20):1909–1917. doi: 10.1056/NEJMoa1007964. 20925534

[39] Chen C, He M, Duan R, Wang F, Liang H, Guan Y, et al. Tu1562: multicenter randomized clinical trial: the impact of short-term proton pump inhibitors verus histamine-2 receptor antagonists on gut microbiota in patients with acute coronary syndrome. Gastroenterology [Internet]. 2022;162(7):S-1010–S-1011. doi: 10.1016/S0016-5085(22)62400-4. PMC11882281 39307932

[40] Kawai T, Oda K, Funao N, Nishimura A, Matsumoto Y, Mizokami Y, Ashida K, Sugano K. Vonoprazan prevents low-dose aspirin-associated ulcer recurrence: randomised phase 3 study. Gut. 2018;67(6):1033–1041. doi: 10.1136/gutjnl-2017-314852. 29196436 PMC5969345

[41] Laine L, DeVault K, Katz P, Mitev S, Lowe J, Hunt B, Spechler S. Vonoprazan versus lansoprazole for healing and maintenance of healing of erosive esophagitis: a randomized trial. Gastroenterology [Internet]. 2023;164(1):61–71. doi: 10.1053/j.gastro.2022.09.041. 36228734

[42] Ashida K, Sakurai Y, Nishimura A, Kudou K, Hiramatsu N, Umegaki E, Iwakiri K, Chiba T. Randomised clinical trial: a dose-ranging study of vonoprazan, a novel potassium-competitive acid blocker, vs. lansoprazole for the treatment of erosive oesophagitis. Aliment Pharmacol Ther. 2015;42(6):685–695. doi: 10.1111/apt.13331. 26201312 PMC5014135

[43] Uemura N, Kinoshita Y, Haruma K, Kushima R, Yao T, Akiyama J, Aoyama N, Baba Y, Suzuki C, Ishiguro K. Vonoprazan as a long-term maintenance treatment for erosive esophagitis: vision, a 5-Year, randomized, open-label study. Clin Gastroenterol Hepatol [Internet]. 2025;23(5):748–757.e5. doi: 10.1016/j.cgh.2024.08.004. 39209187

[44] Wong GLH, Lau LHS, Ching JYL, Tse YK, Ling RHY, Wong VWS, Chiu PWY, Chan FKL. Prevention of recurrent idiopathic gastroduodenal ulcer bleeding: a double-blind, randomised trial. Gut. 2020;69(4):652–657. doi: 10.1136/gutjnl-2019-318715. 31229990

[45] Thorens J, Froehlich F, Schwizer W, Saraga E, Bille J, Gyr K, Duroux P, Nicolet M, Pignatelli B, Blum AL, et al. Bacterial overgrowth during treatment with omeprazole compared with cimetidine: a prospective randomised double blind study. Gut. 1996;39(1):54–59. doi: 10.1136/gut.39.1.54. 8881809 PMC1383231

[46] Ma X, Huang L, Huang Z, Jiang J, Zhao C, Tong H, Feng Z, Gao J, Liu R, Zhang M, et al. The impacts of acid suppression on duodenal microbiota during the early phase of severe acute pancreatitis. Sci Rep [Internet]. 2020;10(1):1–10. doi: 10.1038/s41598-020-77245-1. 33208878 PMC7674417

[47] Vanclooster A, van Deursen C, Jaspers R, Cassiman D, Koek G. Proton pump inhibitors decrease phlebotomy need in HFE hemochromatosis: double-blind randomized placebo-controlled trial. Gastroenterology [Internet]. 2017;153(3):678–680.e2. doi: 10.1053/j.gastro.2017.06.006. 28624580

[48] de Boer WA, Driessen WMM, Jansz AR, Tytgat GNJ. Effect of acid suppression on efficacy of treatment for helicobacter pylori infection. Lancet. 1995;345(8953):817–820. doi: 10.1016/S0140-6736(95)92962-2. 7898228

[49] Graham DY, Tansel A. Interchangeable use of proton pump inhibitors based on relative potency. Clin Gastroenterol Hepatol Off Clin Pract J Am Gastroenterol Assoc. 2018;16(6):800–808.e7. doi: 10.1016/j.cgh.2017.09.033. PMC6913203 28964908

[50] Kirchheiner J, Glatt S, Fuhr U, Klotz U, Meineke I, Seufferlein T, Brockmöller J. Relative potency of proton-pump inhibitors-comparison of effects on intragastric pH. Eur J Clin Pharmacol. 2009;65(1):19–31. doi: 10.1007/s00228-008-0576-5. 18925391

[51] Srinutta T, Chewcharat A, Takkavatakarn K, Praditpornsilpa K, Eiam-Ong S, Jaber BL, Susantitaphong P. Proton pump inhibitors and hypomagnesemia: a meta-analysis of observational studies. Medicine. 2019;98(44):e17788. doi: 10.1097/MD.0000000000017788. 31689852 PMC6946416

[52] Cheungpasitporn W, Thongprayoon C, Kittanamongkolchai W, Srivali N, Edmonds PJ, Ungprasert P, O'Corragain OA, Korpaisarn S, Erickson SB. Proton pump inhibitors linked to hypomagnesemia: a systematic review and meta-analysis of observational studies. Ren Fail. 2015;37(7):1237–1241. doi: 10.3109/0886022X.2015.1057800. 26108134

[53] Choudhury A, Jena A, Jearth V, Dutta AK, Makharia G, Dutta U, Goenka M, Kochhar R, Sharma V. Vitamin B12 deficiency and use of proton pump inhibitors: a systematic review and meta-analysis. Expert Rev Gastroenterol Hepatol. 2023;17(5):479–487. doi: 10.1080/17474124.2023.2204229. 37060552

[54] Laine L, Ahnen D, McClain C, Solcia E, Walsh JH. Review article: potential gastrointestinal effects of long-term acid suppression with proton pump inhibitors. Aliment Pharmacol Ther. 2000;14(6):651–668. doi: 10.1046/j.1365-2036.2000.00768.x. 10848649

[55] Horwitz RI, Feinstein AR. The problem of “protopathic bias” in case-control studies. Am J Med. 1980;68(2):255–258. doi: 10.1016/0002-9343(80)90363-0. 7355896

[56] Bosco JLF, Silliman RA, Thwin SS, Geiger AM, Buist DSM, Prout MN, Yood MU, Haque R, Wei F, Lash TL. A most stubborn bias: no adjustment method fully resolves confounding by indication in observational studies. J Clin Epidemiol. 2010;63(1):64–74. doi: 10.1016/j.jclinepi.2009.03.001. 19457638 PMC2789188

[57] Grimes DA, Schulz KF. False alarms and pseudo-epidemics: the limitations of observational epidemiology. Obstet Gynecol. 2012;120(4):920–927. doi: 10.1097/AOG.0b013e31826af61a. 22996110

[58] Kwon JH, Olsen MA, Dubberke ER. The morbidity, mortality, and costs associated with *Clostridium difficile* infection. Infect Dis Clin N Am. 2015;29(1):123–134. doi: 10.1016/j.idc.2014.11.003. 25677706

[59] Arriola V, Tischendorf J, Musuuza J, Barker A, Rozelle JW, Safdar N. Assessing the risk of hospital-acquired *Clostridium difficile* infection with proton pump inhibitor use: a meta-analysis. Infect Control Hosp Epidemiol. 2016;37(12):1408–1417. doi: 10.1017/ice.2016.194. 27677811 PMC5657489

[60] Kwok CS, Arthur AK, Anibueze CI, Singh S, Cavallazzi R, Loke YK. Risk of *Clostridium difficile* infection with acid suppressing drugs and antibiotics: meta-analysis. Am J Gastroenterol. 2012;107(7):1011–1019. doi: 10.1038/ajg.2012.108. 22525304

[61] Deshpande A, Pant C, Pasupuleti V, Rolston DDK, Jain A, Deshpande N, Thota P, Sferra TJ, Hernandez AV. Association between proton pump inhibitor therapy and *Clostridium difficile* infection in a meta-analysis. Clin Gastroenterol Hepatol Off Clin Pract J Am Gastroenterol Assoc. 2012;10(3):225–233. doi: 10.1016/j.cgh.2011.09.030. 22019794

[62] Janarthanan S, Ditah I, Adler DG, Ehrinpreis MN. *Clostridium difficile*-associated diarrhea and proton pump inhibitor therapy: a meta-analysis. Am J Gastroenterol. 2012;107(7):1001–1010. doi: 10.1038/ajg.2012.179. 22710578

[63] Azab M, Doo L, Doo DH, Elmofti Y, Ahmed M, Cadavona JJ, Liu XB, Shafi A, Joo MK, Yoo JW. Comparison of the hospital-acquired *Clostridium difficile* infection risk of using proton pump inhibitors versus histamine-2 receptor antagonists for prophylaxis and treatment of stress ulcers: a systematic review and meta-analysis. Gut Liver. 2017;11(6):781–788. doi: 10.5009/gnl16568. 28506028 PMC5669593

[64] Oshima T, Wu L, Li M, Fukui H, Watari J, Miwa H. Magnitude and direction of the association between *Clostridium difficile* infection and proton pump inhibitors in adults and pediatric patients: a systematic review and meta-analysis. J Gastroenterol. 2018;53(1):84–94. doi: 10.1007/s00535-017-1369-3. 28744822

[65] Leonard J, Marshall JK, Moayyedi P. Systematic review of the risk of enteric infection in patients taking acid suppression. Am J Gastroenterol. 2007;102(9):2047–2056. quiz 2057. doi: 10.1111/j.1572-0241.2007.01275.x. 17509031

[66] Rezaie A, Buresi M, Lembo A, Lin H, McCallum R, Rao S, Schmulson M, Valdovinos M, Zakko S, Pimentel M. Hydrogen and Methane-based breath testing in gastrointestinal disorders: the North American consensus. Am J Gastroenterol. 2017;112(5):775–784. doi: 10.1038/ajg.2017.46. 28323273 PMC5418558

[67] Jacobs C, Coss Adame E, Attaluri A, Valestin J, Rao SSC. Dysmotility and proton pump inhibitor use are independent risk factors for small intestinal bacterial and/or fungal overgrowth. Aliment Pharmacol Ther. 2013;37(11):1103–1111. doi: 10.1111/apt.12304. 23574267 PMC3764612

[68] Weitsman S, Leite G, Celly S, Morales W, Sanchez M, Parodi G, Villanueva-Millan MJ, Sedighi R, Chang C, Mathur R, et al. A large scale evaluation of the small intestinal microbiome in subjects on proton pump inhibitors. Gastroenterology. 2019;156:S-206. doi: 10.1016/S0016-5085(19)37311-1.

[69] Hegyi P, Petersen OH, Holgate S, Erőss B, Garami A, Szakács Z, Dobszai D, Balaskó M, Kemény L, Peng S, et al. Academia Europaea position paper on translational medicine: the cycle model for translating scientific results into community benefits. J Clin Med. 2020 May;9(5):1532. doi: 10.3390/jcm9051532. 32438747 PMC7290380

[70] Hegyi P, Erőss B, Izbéki F, Párniczky A, Szentesi A. Accelerating the translational medicine cycle: the Academia Europaea pilot. Nat Med. 2021;27:1317–1319. doi: 10.1038/s41591-021-01458-8. 34312557

