# Supplementary material for: Proton pump inhibitors are not associated with an increased risk of Clostridioides difficile infection: a systematic review and meta-analysis of randomized controlled trials
Source: Gut Microbes. 2025 Oct 5;17(1):2562341. doi: 10.1080/19490976.2025.2562341 (PMC12502825; doi:10.1080/19490976.2025.2562341)
Supplement: SUPPLEMENTARY MATERIAL [file KGMI_A_2562341_SM8430.docx]

**Supplementary Material**

**TITLE**

**Proton Pump Inhibitors Are Not Associated With an Increased Risk of *Clostridioides difficile* Infection: A Systematic Review and Meta-analysis of Randomized Controlled Trials**

**AUTHORS**

Diana-Elena Floria^1,2^, Mahmoud Obeidat^1^, Szilárd Váncsa^1,3^, Sarolta Beáta Kávási^1,4^, László Földvári-Nagy^1,5^, Péter Hegyi^1,3,6^, Daniel Sandor Veres^1,7^, Vasile-Liviu Drug^1,2^, Erőss Bálint^1,3,6^

**AFFILIATIONS**

1. Centre for Translational Medicine, Semmelweis University, Budapest, Hungary
2. Grigore T. Popa University of Medicine and Pharmacy Iași, Romania
3. Institute of Pancreatic Diseases, Semmelweis University, Budapest, Hungary
4. Department of Surgery, Toldy Ferenc Hospital, Cegléd, Hungary
5. Department of Morphology and Physiology, Faculty of Health Sciences, Semmelweis University, Budapest, Hungary
6. Institute for Translational Medicine, Medical School, University of Pécs, Pécs, Hungary
7. Department of Biophysics and Radiation Biology, Semmelweis University, Budapest, Hungary

**CORRESPONDING AUTHOR**

Bálint Erőss MD, PhD, FRCP (London)

Centre for Translational Medicine

Semmelweis University, Üllői út 26, Budapest, H-1085, Hungary

E-mail: dr.eross.balint@gmail.com

**Figure and Table legends**

**Table S1.** PRISMA Checklist.

**Table S2.** Detailed search key.

**Table S3.** Criteria used for ascertaining *Clostridioides difficile* infection in included studies.

**Figure S1.** Forest plot showing the risk of developing *Clostridioides difficile* infection in patients receiving proton pump inhibitors compared to placebo: Subgroup analysis based on patient population – hospitalised vs. outpatients.

**Figure S2.** Forest plot showing the risk of developing *Clostridioides difficile* infection in patients receiving proton pump inhibitors compared to placebo: Subgroup analysis based on treatment duration – long- vs. short-term therapy.

**Figure S3.** Forest plot showing the risk of developing *Clostridioides difficile* infection in patients receiving proton pump inhibitors compared to placebo: Subgroup analysis based on duration of follow-up – long- vs. medium vs. short-term follow-up.

**Figure S4.** Forest plot showing the risk of developing *Clostridioides difficile* infection in patients receiving proton pump inhibitors compared to H2-blockers: Subgroup analysis based on patient population – hospitalised vs. outpatients.

**Figure S5.** Forest plot showing the risk of developing *Clostridioides difficile* infection in patients receiving proton pump inhibitors compared to H2-blockers: Subgroup analysis based on treatment duration – long- vs. short-term therapy.

**Figure S6.** Forest plot showing the risk of developing *Clostridioides difficile* infection in patients receiving proton pump inhibitors compared to H2-blocker: Subgroup analysis based on duration of follow-up – long- vs. medium vs. short-term follow-up.

**Figure S7.** Detailed assessment of the risk of bias for *Clostridioides difficile* infection.

**Figure S8.** Detailed assessment of the risk of bias for other enteric infections.

**Figure S9**. Detailed assessment of the risk of bias for SIBO.

**Table S4, S5.** Certainty of evidence assessments.

**Figure S10.** Funnel plot for publication bias assessment.

**Table S1.** PRISMA checklist

| **Section and Topic** | **Item #** | **Checklist item** | **Location where item is reported** |
| --- | --- | --- | --- |
| **TITLE** | | |  |
| Title | 1 | Identify the report as a systematic review. | Title – page 1 |
| **ABSTRACT** | | |  |
| Abstract | 2 | See the PRISMA 2020 for Abstracts checklist. | Page 3 |
| **INTRODUCTION** | | |  |
| Rationale | 3 | Describe the rationale for the review in the context of existing knowledge. | Introduction – page 4 |
| Objectives | 4 | Provide an explicit statement of the objective(s) or question(s) the review addresses. | Introduction – page 4 |
| **METHODS** | | |  |
| Eligibility criteria | 5 | Specify the inclusion and exclusion criteria for the review and how studies were grouped for the syntheses. | Methods – page 4 (Eligibility criteria) |
| Information sources | 6 | Specify all databases, registers, websites, organisations, reference lists and other sources searched or consulted to identify studies. Specify the date when each source was last searched or consulted. | Methods – page 4 (Information sources) |
| Search strategy | 7 | Present the full search strategies for all databases, registers and websites, including any filters and limits used. | Supplementary Material – Table S2 |
| Selection process | 8 | Specify the methods used to decide whether a study met the inclusion criteria of the review, including how many reviewers screened each record and each report retrieved, whether they worked independently, and if applicable, details of automation tools used in the process. | Methods – page 5 (Selection process) |
| Data collection process | 9 | Specify the methods used to collect data from reports, including how many reviewers collected data from each report, whether they worked independently, any processes for obtaining or confirming data from study investigators, and if applicable, details of automation tools used in the process. | Methods – page 5 (Data collection process) |
| Data items | 10a | List and define all outcomes for which data were sought. Specify whether all results that were compatible with each outcome domain in each study were sought (e.g. for all measures, time points, analyses), and if not, the methods used to decide which results to collect. | Methods – page 5 (Data items) |
|  | 10b | List and define all other variables for which data were sought (e.g. participant and intervention characteristics, funding sources). Describe any assumptions made about any missing or unclear information. | Methods – page 5 (Data items) |
| Study risk of bias assessment | 11 | Specify the methods used to assess risk of bias in the included studies, including details of the tool(s) used, how many reviewers assessed each study and whether they worked independently, and if applicable, details of automation tools used in the process. | Methods – page 5 (Risk of bias and quality of evidence assessment) |
| Effect measures | 12 | Specify for each outcome the effect measure(s) (e.g. risk ratio, mean difference) used in the synthesis or presentation of results. | Methods – page 6 (Synthesis methods) |
| Synthesis methods | 13a | Describe the processes used to decide which studies were eligible for each synthesis (e.g. tabulating the study intervention characteristics and comparing against the planned groups for each synthesis (item #5)). | Methods – page 6 (Synthesis methods); Supplementary Material - Statistical Methods |
|  | 13b | Describe any methods required to prepare the data for presentation or synthesis, such as handling of missing summary statistics, or data conversions. | Methods – page 6 (Synthesis methods); Supplementary Material - Statistical Methods |
|  | 13c | Describe any methods used to tabulate or visually display results of individual studies and syntheses. | Methods – page 6 (Synthesis methods) |
|  | 13d | Describe any methods used to synthesize results and provide a rationale for the choice(s). If meta-analysis was performed, describe the model(s), method(s) to identify the presence and extent of statistical heterogeneity, and software package(s) used. | Methods – page 6 (Synthesis methods); Supplementary Material - Statistical Methods |
|  | 13e | Describe any methods used to explore possible causes of heterogeneity among study results (e.g. subgroup analysis, meta-regression). | Methods – page 6 (Synthesis methods); Supplementary Material - Statistical Methods |
|  | 13f | Describe any sensitivity analyses conducted to assess robustness of the synthesized results. | Methods – page 6 (Synthesis methods); Supplementary Material - Statistical Methods |
| Reporting bias assessment | 14 | Describe any methods used to assess risk of bias due to missing results in a synthesis (arising from reporting biases). | Methods – page 5 (Risk of bias and quality of evidence assessment) |
| Certainty assessment | 15 | Describe any methods used to assess certainty (or confidence) in the body of evidence for an outcome. | Methods – page 5 (Risk of bias and quality of evidence assessment) |
| **RESULTS** | | |  |
| Study selection | 16a | Describe the results of the search and selection process, from the number of records identified in the search to the number of studies included in the review, ideally using a flow diagram. | Results – Page 6 (Search and selection); PRISMA flowchart (Figure 1) |
|  | 16b | Cite studies that might appear to meet the inclusion criteria, but which were excluded, and explain why they were excluded. | PRISMA flowchart (Figure 1) |
| Study characteristics | 17 | Cite each included study and present its characteristics. | Table 1 – page 13 |
| Risk of bias in studies | 18 | Present assessments of risk of bias for each included study. | Supplementary Material – Figure S7, S8, S9 |
| Results of individual studies | 19 | For all outcomes, present, for each study: (a) summary statistics for each group (where appropriate) and (b) an effect estimate and its precision (e.g. confidence/credible interval), ideally using structured tables or plots. | Results – page 7 (Quantitative synthesis); Figure 2, Figure 3 |
| Results of syntheses | 20a | For each synthesis, briefly summarise the characteristics and risk of bias among contributing studies. | Results – page 7 (Risk of bias assessment) |
|  | 20b | Present results of all statistical syntheses conducted. If meta-analysis was done, present for each the summary estimate and its precision (e.g. confidence/credible interval) and measures of statistical heterogeneity. If comparing groups, describe the direction of the effect. | Results – page 7 (Quantitative synthesis); Figure 2, Figure 3 |
|  | 20c | Present results of all investigations of possible causes of heterogeneity among study results. | Supplementary Material – Results (Figure S1, S2, S3, S4, S5, S6, S7, S8) |
|  | 20d | Present results of all sensitivity analyses conducted to assess the robustness of the synthesized results. | NA |
| Reporting biases | 21 | Present assessments of risk of bias due to missing results (arising from reporting biases) for each synthesis assessed. | Results – Page 8 – Risk of Bias assessment; Supplementary Material – Figure S7, S8, S9 |
| Certainty of evidence | 22 | Present assessments of certainty (or confidence) in the body of evidence for each outcome assessed. | Results – Page 8 – Certainty of evidence |
| **DISCUSSION** | | |  |
| Discussion | 23a | Provide a general interpretation of the results in the context of other evidence. | Discussion – page 9 |
|  | 23b | Discuss any limitations of the evidence included in the review. | Discussion – page 10 (Strengths and limitations) |
|  | 23c | Discuss any limitations of the review processes used. | Discussion – page 10 (Strengths and limitations) |
|  | 23d | Discuss implications of the results for practice, policy, and future research. | Discussion – page 10 (Implications for practice and research) |
| **OTHER INFORMATION** | | |  |
| Registration and protocol | 24a | Provide registration information for the review, including register name and registration number, or state that the review was not registered. | Methods – page 4 |
|  | 24b | Indicate where the review protocol can be accessed, or state that a protocol was not prepared. | Methods – page 4 |
|  | 24c | Describe and explain any amendments to information provided at registration or in the protocol. | Methods – page 4 |
| Support | 25 | Describe sources of financial or non-financial support for the review, and the role of the funders or sponsors in the review. | Page 2 - Funding |
| Competing interests | 26 | Declare any competing interests of review authors. | Page 2 – Conflicts of interest |
| Availability of data, code and other materials | 27 | Report which of the following are publicly available and where they can be found: template data collection forms; data extracted from included studies; data used for all analyses; analytic code; any other materials used in the review. | Page 2 – Data availability |

*From:*  Page MJ, McKenzie JE, Bossuyt PM, Boutron I, Hoffmann TC, Mulrow CD, et al. The PRISMA 2020 statement: an updated guideline for reporting systematic reviews. BMJ 2021;372:n71. doi: 10.1136/bmj.n71

**Table S2.** Detailed search key

| **Database** | **Searchkey** |
| --- | --- |
| **PubMed** | ((pump inhibitor) OR PPI OR rabeprazole OR lansoprazole OR dexlansoprazole OR pantoprazole OR esomeprazole OR omeprazole OR tenatoprazole OR ilaprazole)  AND random* |
| **Embase** | ((pump inhibitor) OR PPI OR rabeprazole OR lansoprazole OR dexlansoprazole OR pantoprazole OR esomeprazole OR omeprazole OR tenatoprazole OR ilaprazole)  AND random* |
| **CENTRAL** | ((pump inhibitor) OR PPI OR rabeprazole OR lansoprazole OR dexlansoprazole OR pantoprazole OR esomeprazole OR omeprazole OR tenatoprazole OR ilaprazole)  AND random* |

**Table S3.** Criteria used for ascertaining *Clostridioides difficile* infection in included studies.

| **Author, year** | Definition |
| --- | --- |
| **Alhazzani et al., 2017(1)** | *Clostridioides difficile* toxin–positive stool or colonoscopic or histopathologic findings demonstrating pseudomembranous colitis |
| **Bhatt et al. 2010(2)** | Not specified |
| **Chen et al., 2022 (ABSTRACT)(3)** | Not specified |
| **Cook et al., 2024**(4) | Microbiological evidence of toxin-producing *Clostridioides difficile* or pseudomembranous colitis on colonoscopy. |
| **De Boer et al., 1995**(5) | Pseudomembranous colitis – possibly colonoscopy-based |
| **El-Kersh et al., 2018**(6) | Positive toxin assay |
| **Kawai et al., 2017**(7) | *Clostridia* test positive |
| **Krag et al., 2018**(8) | Treatment with antibiotics (enteral vancomycin, intravenous or enteral metronidazole, enteral fidaxomicin) for suspected or proven *Clostridium difficile* infection |
| **Laine et al., 2023**(9) | Not specified |
| **Moayyedi et al., 2019**(10) | Not specified |
| **Selvanderan et al., 2016**(11) | *Clostridium difficile* toxin DNA polymerase chain reaction testing |
| **Uemura et al., 2025**(12) | Not specified |
| **Wee et al., 2013 (ABSTRACT)**(13) | Not specified |
| **Wong et al., 2020**(14) | Not specified |
| **Young et al., 2020**(15) | Toxin or culture-positive stool sample |

**Statistical Methods**

We provide the following additional details regarding data synthesis:

For pooling the effect sizes, pooled Risk Ratios (RR) were calculated by the Mantel-Haenszel method (16,17). The exact Mantel-Haenszel method (without continuity correction) was used in order to handle zero cell counts, as recommended by Cooper and Sweeting (18,19). A Hartung-Knapp adjustment (20,21) was used for the CIs. This adjustment was applied only if it provided a more conservative estimation than the classical approach, as recommended by Jackson et al. (22).

To estimate the heterogeneity variance measure (τ^2^), the Paule-Mandel method (23) was used, with the Q profile method for CI (as recommended by Harrer et al. and Veroniki et al. (24,25)).

Pooled risks in the experimental group and control group were calculated using a random intercept logistic regression model with maximum-likelihood estimator for τ^2^ and t-distribution based CI.

In case of zero cell counts, individual study RR with 95% CI were calculated by adding 0.5 as continuity correction. This was used only for visualization on the forest plots.

For subgroup analyses we used a fixed-effects “plural” model (mixed-effects model). We assumed that all subgroups share a common τ^2^ value, as we did not anticipate differences in the between-study heterogeneity in the subgroups and the study number was relatively low in some subgroup (recommended by Borenstein et al. (26)). We planned to assess the between-subgroup differences using a “Cochrane Q” test (an omnibus test) (24) if at least 5 studies were included in each subgroup. The null hypothesis was rejected on a 5% significance level. We reported the subgroup pooled estimates, if at least 3 studies were available in the subgroup.


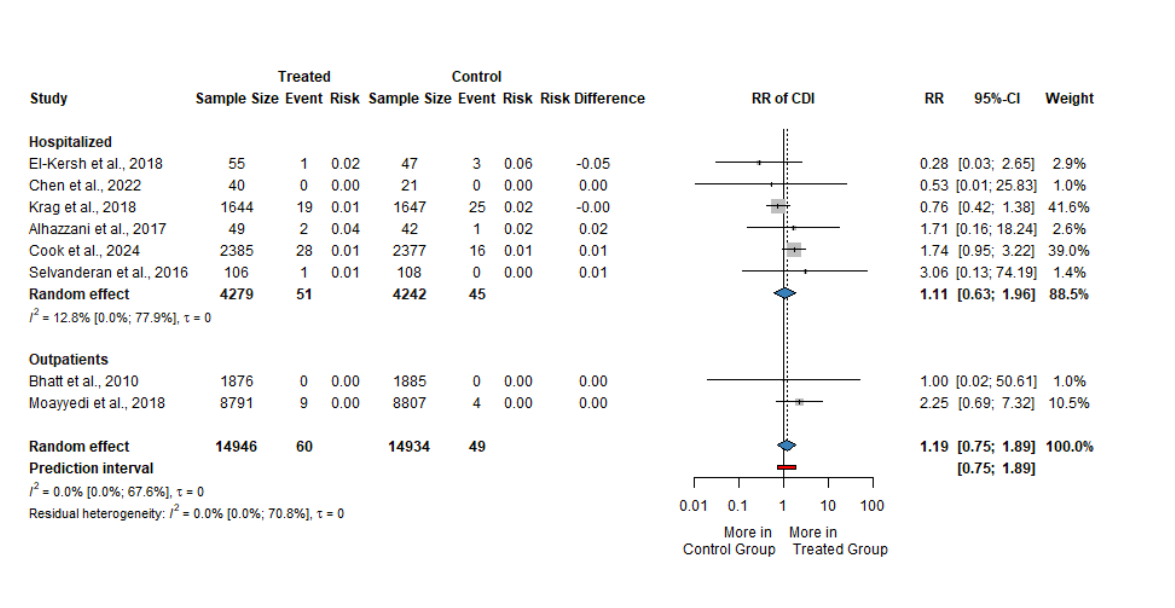

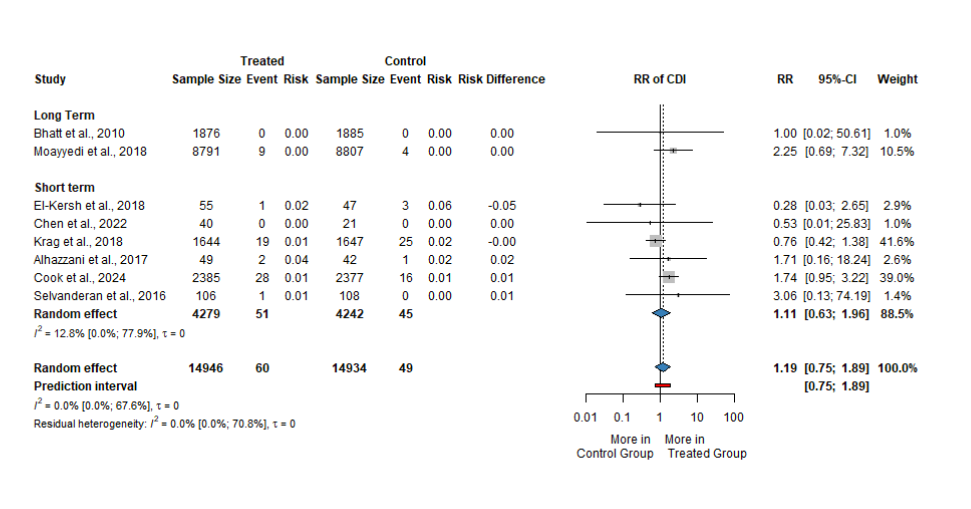


**Figure S1.** Forest plot showing the risk of developing *Clostridioides difficile* infection in patients receiving proton pump inhibitors compared to placebo: Subgroup analysis based on patient population – hospitalised vs. outpatients (*RR, risk ratio; CI, confidence interval; CDI, Clostridioides difficile infection*).

**Figure S2.** Forest plot showing the risk of developing *Clostridioides difficile* infection in patients receiving proton pump inhibitors compared to placebo: Subgroup analysis based on treatment duration – long- vs. short-term therapy (*RR, risk ratio; CI, confidence interval; CDI, Clostridioides difficile infection*).


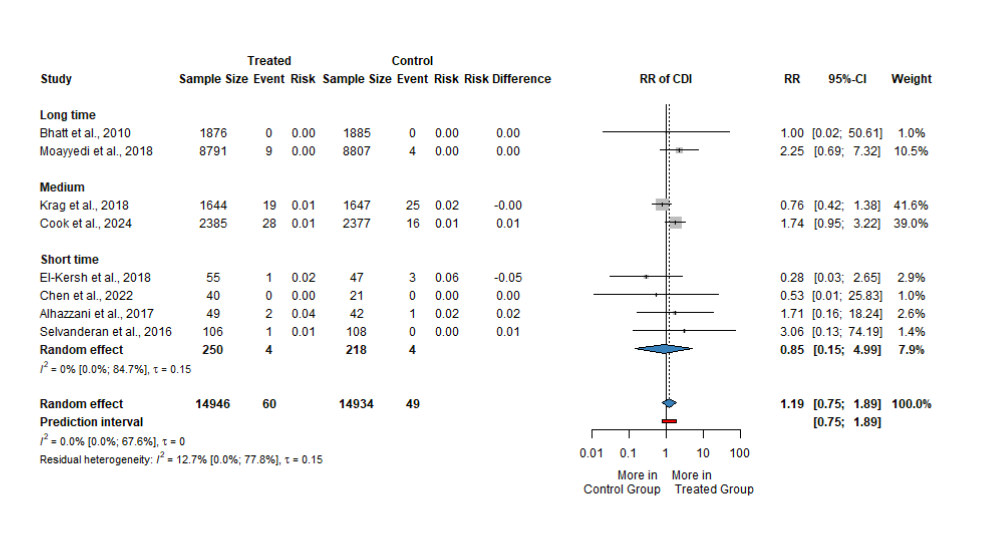


**Figure S3.** Forest plot showing the risk of developing *Clostridioides difficile* infection in patients receiving proton pump inhibitors compared to placebo: Subgroup analysis based on duration of follow-up – long- vs. medium vs. short-term follow-up (short term defined as only for the duration of hospital stay or less than a week; medium defined as up to 90 days; *RR, risk ratio; CI, confidence interval; CDI, Clostridioides difficile infection*).

**
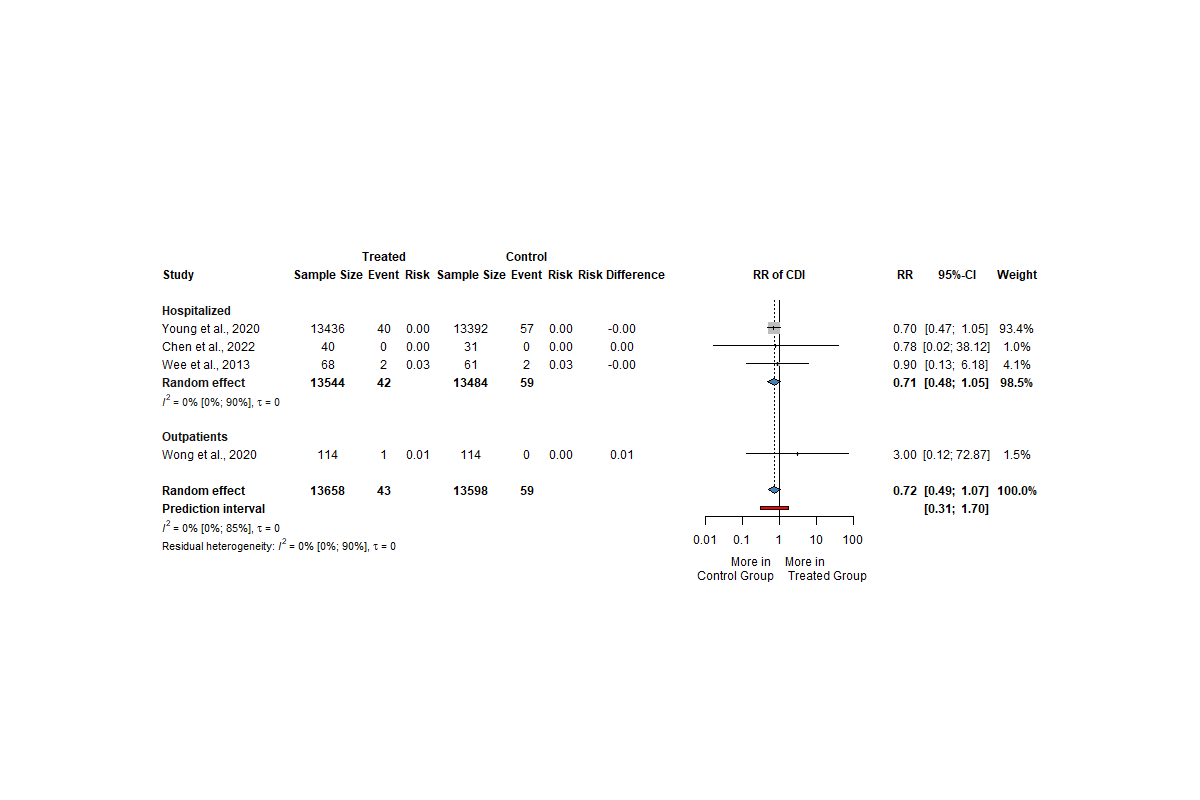
**

**Figure S4.** Forest plot showing the risk of developing Clostridioides difficile infection in patients receiving proton pump inhibitors compared to H2-blockers: Subgroup analysis based on patient population – hospitalised vs. outpatients (RR, risk ratio; CI, confidence interval; CDI, Clostridioides difficile infection).

**
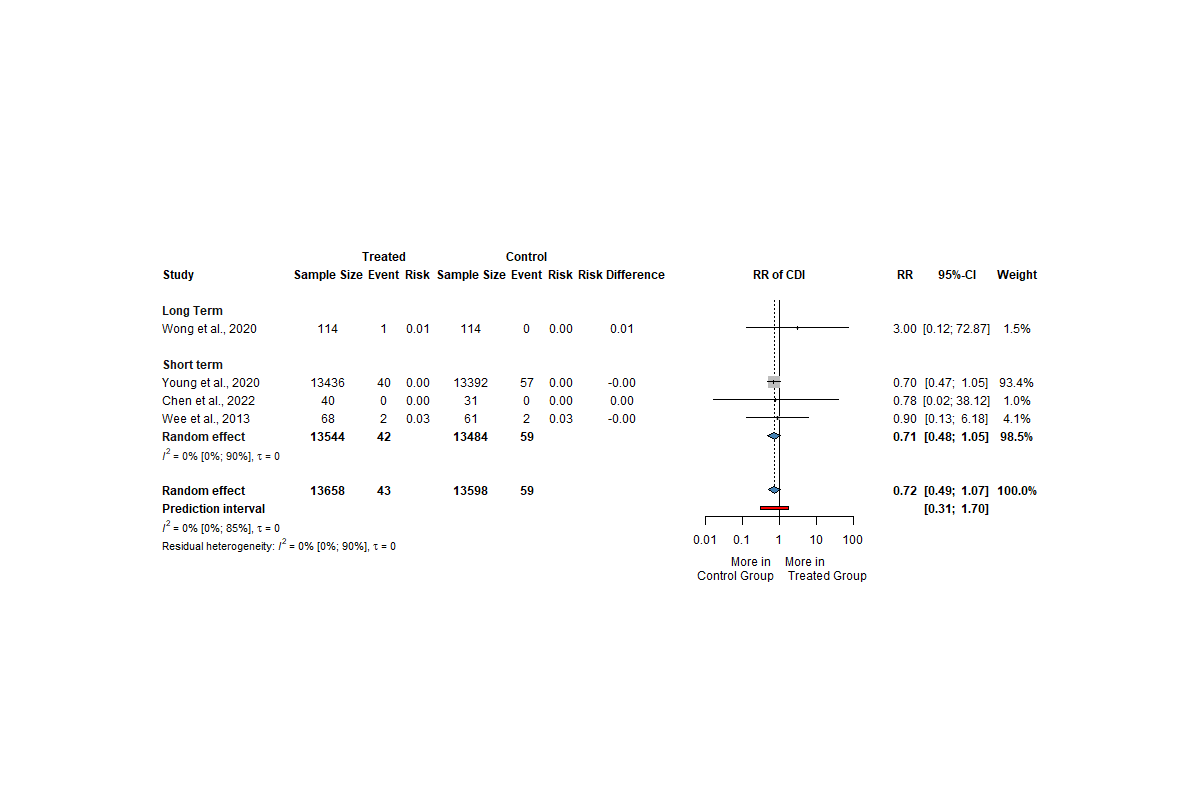
**

**Figure S5.** Forest plot showing the risk of developing *Clostridioides difficile* infection in patients receiving proton pump inhibitors compared to H2-blockers: Subgroup analysis based on treatment duration – long- vs. short-term therapy (*RR, risk ratio; CI, confidence interval; CDI, Clostridioides difficile infection*).

**
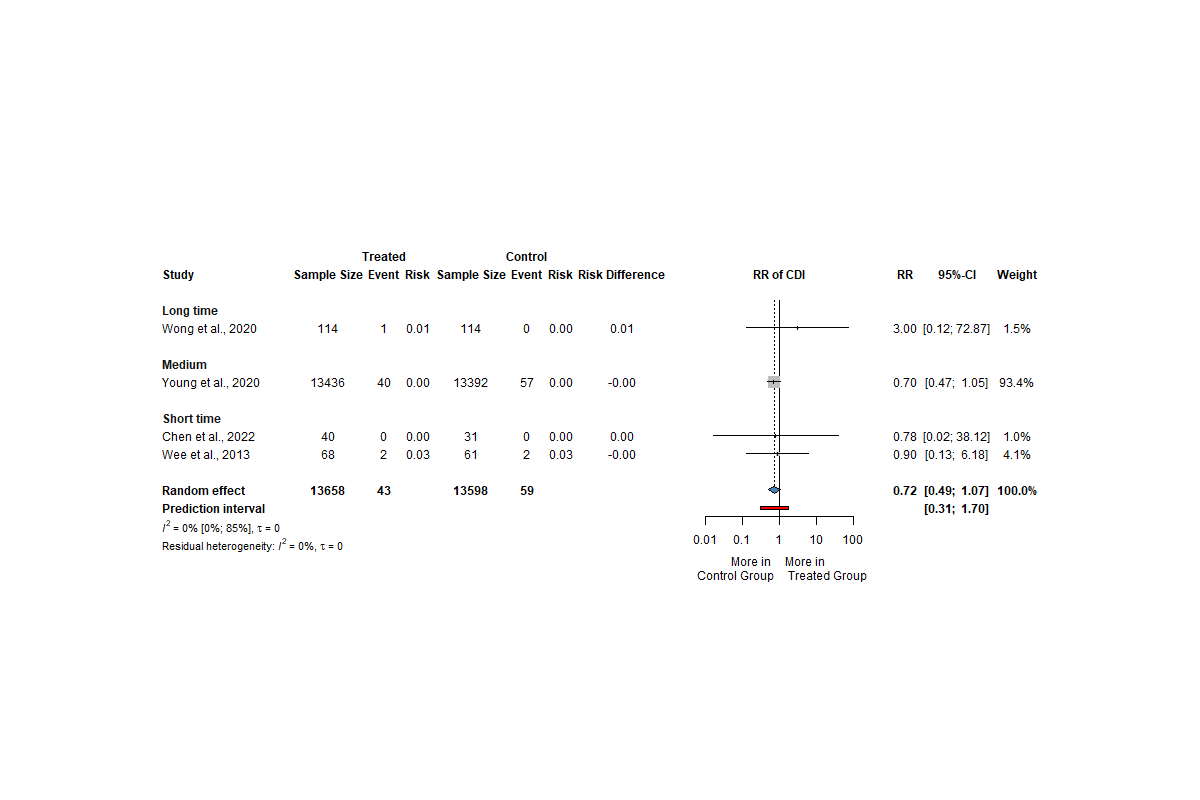
**

**Figure S6.** Forest plot showing the risk of developing *Clostridioides difficile* infection in patients receiving proton pump inhibitors compared to H2-blocker: Subgroup analysis based on duration of follow-up – long- vs. medium vs. short-term follow-up (short term defined as only for the duration of hospital stay or less than a week; medium defined as up to 90 days; *RR, risk ratio; CI, confidence interval; CDI, Clostridioides difficile infection*).


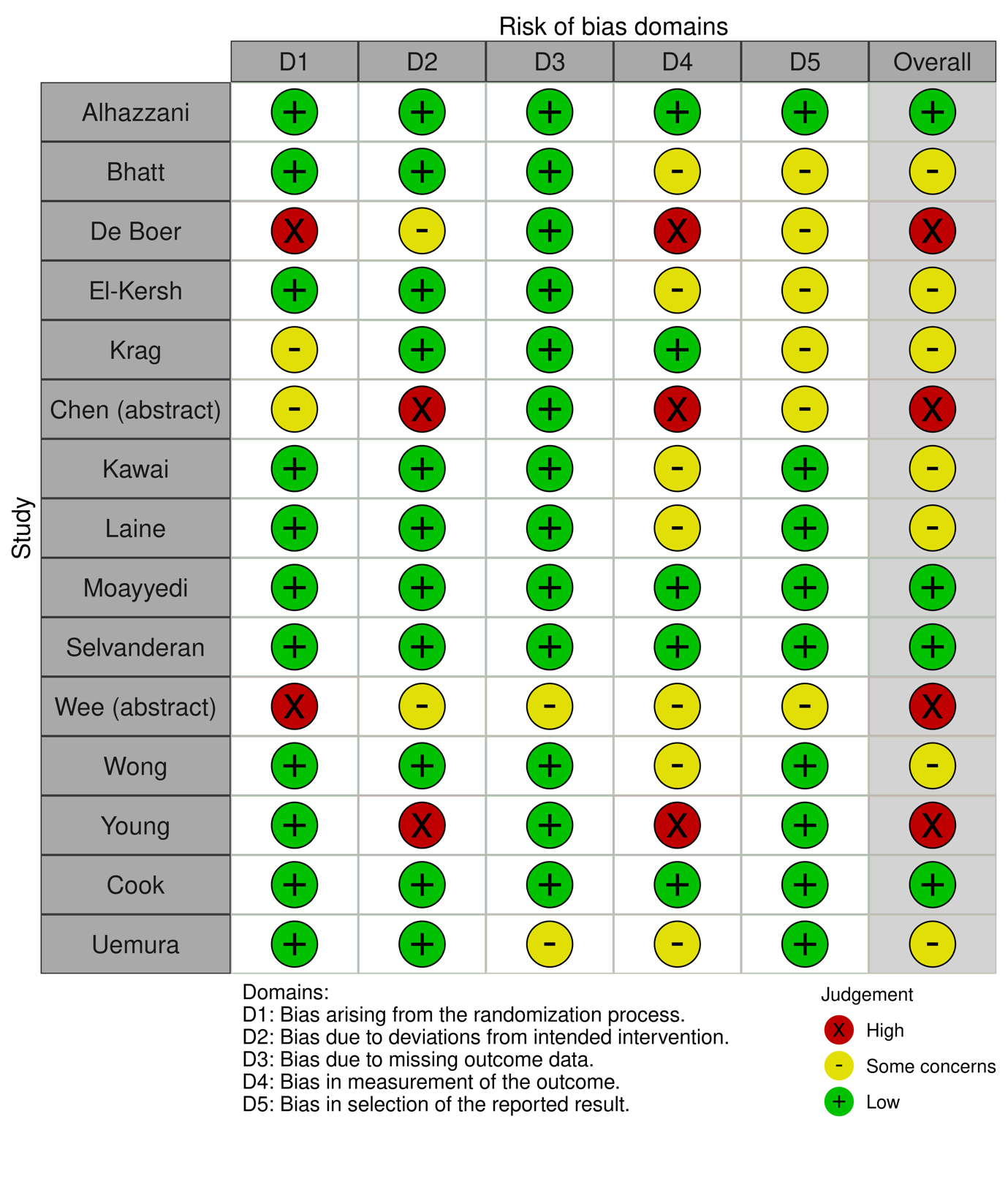


**Figure S7.** Detailed assessment of the risk of bias for *Clostridioides difficile* infection.


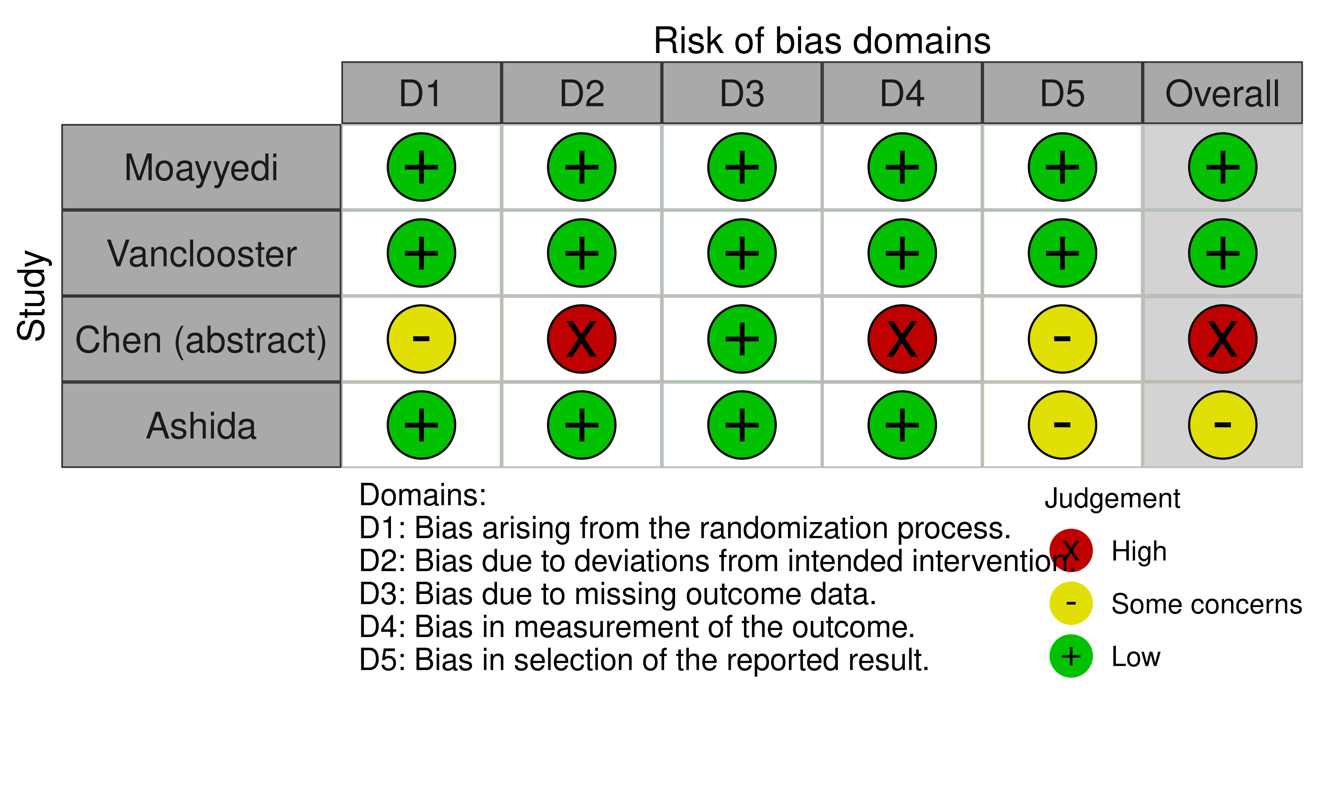


**Figure S8.** Detailed assessment of the risk of bias for other enteric infections.


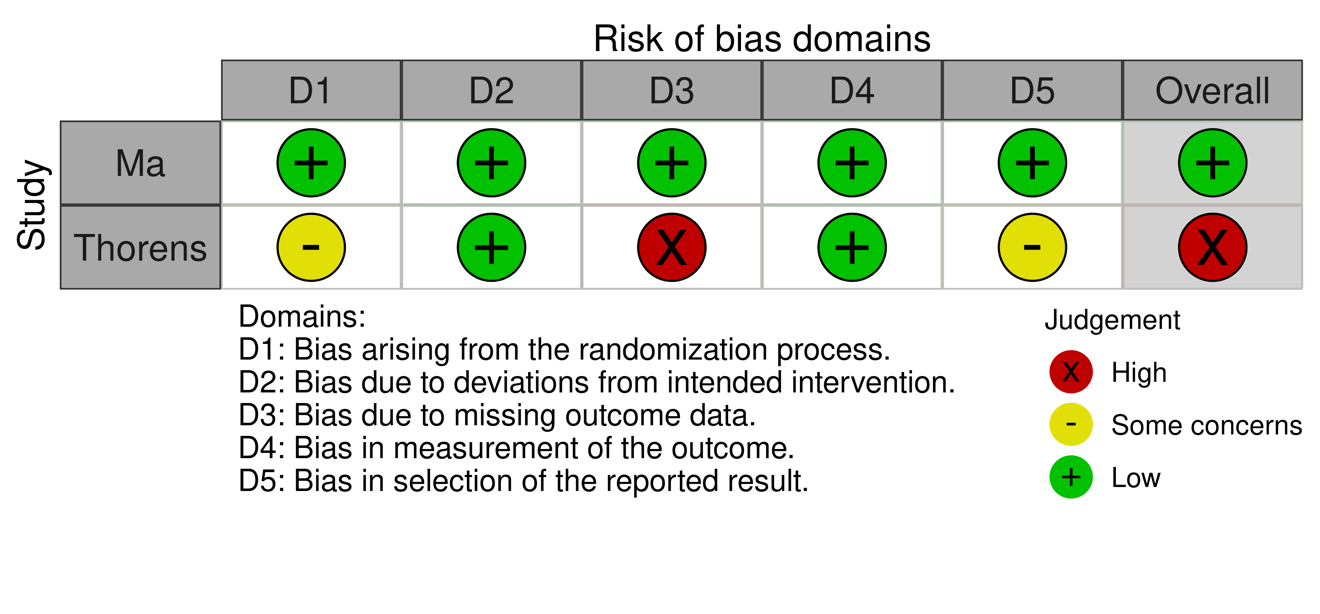


**Figure S9.** Detailed assessment of the risk of bias for SIBO.

**Certainty of Evidence**

**Table S4: Do** Proton Pump Inhibitors increase the risk of *Clostridioides difficile* infection compared to Placebo in Adults?

| **Certainty assessment** | | | | | | | **№ of patients** | | **Effect** | | **Certainty** | **Importance** |
| --- | --- | --- | --- | --- | --- | --- | --- | --- | --- | --- | --- | --- |
| **№ of studies** | **Study design** | **Risk of bias** | **Inconsistency** | **Indirectness** | **Imprecision** | **Other considerations** | **Proton Pump Inhibitors** | **Placebo** | **Relative (95% CI)** | **Absolute (95% CI)** |  |  |
| **Clostridioides difficile Infection** | | | | | | | | | | | | |
| 8 | randomised trials | serious^a^ | not serious^b^ | very serious^c^ | not serious | none | 60/14946 (0.4%) | 49/14934 (0.3%) | **RR 1.19** (0.75 to 1.89) | **1 more per 1,000** (from 1 fewer to 3 more) | ⨁◯◯◯ Very low^a,b,c^ | IMPORTANT |

**CI:** confidence interval; **RR:** risk ratio

#### Explanations

a. In most of the studies that reported this outcome the risk of bias was deemed moderate or high; therefore, the authors chose to downgrade the level of evidence. Most of the concern for bias arose from the fourth and fifth domains of the RoB tool (as the way of ascertaining a diagnosis of CDI was not clearly described in most studies, while many of them also did not have a pre-specified statistical analysis plan available).

b. There was no unexplained heterogeneity in our results.

c. The patient categories in the included studies varied widely, from hospitalized subjects to outpatients, with different indications for acid-suppressive treatment. The included studies also used varying doses, ways of administration and durations of treatment with Proton Pump Inhibitors. Additionally, the time of follow-up ranged from period of hospital stay to 3 years of follow-up. Therefore, the authors chose to downgrade the level of evidence.

**Table S5: Do** Proton Pump Inhibitors increase the risk of *Clostridioides difficile* infection compared to H2 Receptor Antagonists in Adults?

| **Certainty assessment** | | | | | | | **№ of patients** | | **Effect** | | **Certainty** | **Importance** |
| --- | --- | --- | --- | --- | --- | --- | --- | --- | --- | --- | --- | --- |
| **№ of studies** | **Study design** | **Risk of bias** | **Inconsistency** | **Indirectness** | **Imprecision** | **Other considerations** | **Proton Pump Inhibitors** | **H2 Receptor Antagonists** | **Relative (95% CI)** | **Absolute (95% CI)** |  |  |
| **Clostridioides difficile Infection** | | | | | | | | | | | | |
| 4 | randomised trials | serious^a^ | not serious^b^ | very serious^c^ | not serious | none | 43/13658 (0.3%) | 59/13598 (0.4%) | **RR 0.72** (0.49 to 1.07) | **1 fewer per 1,000** (from 2 fewer to 0 fewer) | ⨁◯◯◯ Very low | IMPORTANT |

**CI:** confidence interval; **RR:** risk ratio

#### Explanations

a. In most of the studies that reported this outcome the risk of bias was deemed moderate or high; therefore, the authors chose to downgrade the level of evidence. Most of the concern for bias arose from the fourth and fifth domains of the RoB tool (as the way of ascertaining a diagnosis of CDI was not clearly described in most studies, while many of them also did not have a pre-specified statistical analysis plan available).

b. The results for this outcome did not present significant unexplained heterogeneity.

c. The patient categories in the included studies varied widely, from hospitalized subjects to outpatients, with different indications for acid-suppressive treatment. The included studies also used varying doses, ways of administration and durations of treatment. Therefore, the authors chose to downgrade the level of evidence.

**
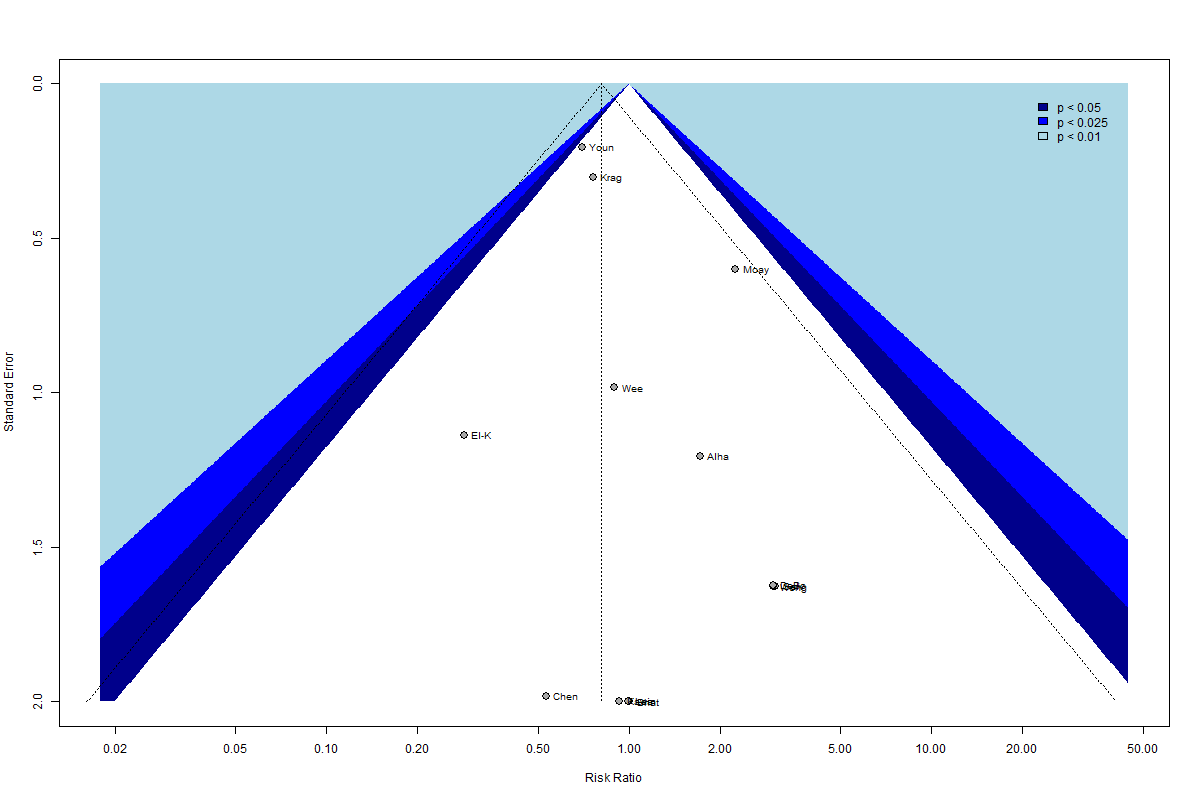
Figure S10.** Funnel plot for publication bias assessment.

**References**

1. Alhazzani W, Guyatt G, Alshahrani M, Deane AM, Marshall JC, Hall R, et al. Withholding Pantoprazole for Stress Ulcer Prophylaxis in Critically Ill Patients: A Pilot Randomized Clinical Trial and Meta-Analysis∗. Crit Care Med. 2017;45(7):1121–9.

2. Bhatt DL, Cryer BL, Contant CF, Cohen M, Lanas A, Schnitzer TJ, et al. Clopidogrel with or without Omeprazole in Coronary Artery Disease. N Engl J Med. 2010;363(20):1909–17.

3. Chen C, He M, Duan R, Wang F, Liang H, Guan Y, et al. Tu1562: MULTICENTER RANDOMIZED CLINICAL TRIAL: THE IMPACT OF SHORT-TERM PROTON PUMP INHIBITORS VERUS HISTAMINE-2 RECEPTOR ANTAGONISTS ON GUT MICROBIOTA IN PATIENTS WITH ACUTE CORONARY SYNDROME. Gastroenterology [Internet]. 2022;162(7):S-1010-S-1011. Available from: http://dx.doi.org/10.1016/S0016-5085(22)62400-4

4. Cook D, Deane A, Lauzier F, Zytaruk N, Guyatt G, Saunders L, et al. Stress Ulcer Prophylaxis during Invasive Mechanical Ventilation. N Engl J Med. 2024;391(1):9–20.

5. de Boer WA, Driessen WMM, Jansz AR, Tytgat GNJ. Effect of acid suppression on efficacy of treatment for Helicobacter pylori infection. Lancet. 1995;345(8953):817–20.

6. El-Kersh K, Jalil B, McClave SA, Cavallazzi R, Guardiola J, Guilkey K, et al. Enteral nutrition as stress ulcer prophylaxis in critically ill patients: A randomized controlled exploratory study. J Crit Care [Internet]. 2018;43:108–13. Available from: https://doi.org/10.1016/j.jcrc.2017.08.036

7. Kawai T, Oda K, Funao N, Nishimura A, Matsumoto Y, Mizokami Y, et al. Vonoprazan prevents low-dose aspirin-associated ulcer recurrence: Randomised phase 3 study. Gut. 2018;67(6):1033–41.

8. Krag M, Marker S, Perner A, Wetterslev J, Wise MP, Schefold JC, et al. Pantoprazole in Patients at Risk for Gastrointestinal Bleeding in the ICU. N Engl J Med. 2018;379(23):2199–208.

9. Laine L, DeVault K, Katz P, Mitev S, Lowe J, Hunt B, et al. Vonoprazan Versus Lansoprazole for Healing and Maintenance of Healing of Erosive Esophagitis: A Randomized Trial. Gastroenterology [Internet]. 2023;164(1):61–71. Available from: https://doi.org/10.1053/j.gastro.2022.09.041

10. Moayyedi P, Eikelboom JW, Bosch J, Connolly SJ, Dyal L, Shestakovska O, et al. Safety of Proton Pump Inhibitors Based on a Large, Multi-Year, Randomized Trial of Patients Receiving Rivaroxaban or Aspirin. Gastroenterology. 2019;157(3):682-691.e2.

11. Selvanderan SP, Summers MJ, Finnis ME, Plummer MP, Ali Abdelhamid Y, Anderson MB, et al. Pantoprazole or Placebo for Stress Ulcer Prophylaxis (POP-UP): Randomized Double-Blind Exploratory Study. Crit Care Med. 2016;44(10):1842–50.

12. Uemura N, Kinoshita Y, Haruma K, Kushima R, Yao T, Akiyama J, et al. Vonoprazan as a Long-Term Maintenance Treatment for Erosive Esophagitis: VISION, a 5-Year, Randomized, Open-Label Study. Clin Gastroenterol Hepatol [Internet]. 2025;23(5):748-757.e5. Available from: https://doi.org/10.1016/j.cgh.2024.08.004

13. Wee B, Liu C, Cohen H, Kravchuk S, Reddy K. IV famotidine vs. IV pantoprazole for stress ulcer prevention in the ICU: A prospective study. Crit Care Med [Internet]. 2013;41(12 SUPPL. 1):A181. Available from: http://ovidsp.ovid.com/ovidweb.cgi?T=JS&PAGE=reference&D=emed11&NEWS=N&AN=71533909

14. Wong GLH, Lau LHS, Ching JYL, Tse YK, Ling RHY, Wong VWS, et al. Prevention of recurrent idiopathic gastroduodenal ulcer bleeding: A double-blind, randomised trial. Gut. 2020;69(4):652–7.

15. Young PJ, Bagshaw SM, Forbes AB, Nichol AD, Wright SE, Bailey M, et al. Effect of Stress Ulcer Prophylaxis with Proton Pump Inhibitors vs Histamine-2 Receptor Blockers on In-Hospital Mortality among ICU Patients Receiving Invasive Mechanical Ventilation: The PEPTIC Randomized Clinical Trial. JAMA - J Am Med Assoc. 2020;323(7):616–26.

16. MANTEL N, HAENSZEL W. Statistical aspects of the analysis of data from retrospective studies of disease. J Natl Cancer Inst. 1959 Apr;22(4):719–48.

17. Robins J, Greenland S, Breslow NE. A general estimator for the variance of the Mantel-Haenszel odds ratio. Am J Epidemiol. 1986 Nov;124(5):719–23.

18. Cooper H, Hedges L, Valentine J. The handbook of research synthesis and meta-analysis. 2nd ed. New York: Russell Sage Foundation; 2009.

19. Sweeting MJ, Sutton AJ, Lambert PC. What to add to nothing? Use and avoidance of continuity corrections in meta-analysis of sparse data. Stat Med. 2004 May;23(9):1351–75.

20. Knapp G, Hartung J. Improved tests for a random effects meta-regression with a single covariate. Stat Med [Internet]. 2003 Sep 15;22(17):2693–710. Available from: https://onlinelibrary.wiley.com/doi/10.1002/sim.1482

21. IntHout J, Ioannidis JPA, Borm GF. The Hartung-Knapp-Sidik-Jonkman method for random effects meta-analysis is straightforward and considerably outperforms the standard DerSimonian-Laird method. BMC Med Res Methodol [Internet]. 2014;14(1):25. Available from: https://doi.org/10.1186/1471-2288-14-25

22. Jackson D, Law M, Rücker G, Schwarzer G. The Hartung‐Knapp modification for random‐effects meta‐analysis: A useful refinement but are there any residual concerns? Stat Med [Internet]. 2017 Nov 10;36(25):3923–34. Available from: https://onlinelibrary.wiley.com/doi/10.1002/sim.7411

23. Paule RC, Mandel J. Consensus Values and Weighting Factors. J Res Natl Bur Stand. 1982;87(5):377–85.

24. Harrer M, Cuijpers P, Toshi F, Ebert DD. Doing Meta-Analysis with R: A Hands-On Guide. 1st editio. Boca Raton, FL: Chapman and Hall/CRC; 2021.

25. Veroniki AA, Jackson D, Viechtbauer W, Bender R, Bowden J, Knapp G, et al. Methods to estimate the between‐study variance and its uncertainty in meta‐analysis. Res Synth Methods [Internet]. 2016 Mar 2;7(1):55–79. Available from: https://onlinelibrary.wiley.com/doi/10.1002/jrsm.1164

26. Borenstein M, Hedges L, Higgins J, Rothstein H. Introduction to Meta-Analysis. Chichester, UK: John Wiley & Sons, Ltd; 2009.
